# Efficacy of pembrolizumab in advanced cancer of the vulva: a systematic review and single-arm meta-analysis

**DOI:** 10.3389/fonc.2024.1352975

**Published:** 2024-02-19

**Authors:** Roxana Schwab, Lina Judit Schiestl, Lorena Cascant Ortolano, Philip Herbert Klecker, Mona Wanda Schmidt, Katrin Almstedt, Anne-Sophie Heimes, Walburgis Brenner, Kathrin Stewen, Marcus Schmidt, Annette Hasenburg

**Affiliations:** ^1^Department of Obstetrics and Gynecology, University Medical Center, Johannes Gutenberg University Mainz, Mainz, Germany; ^2^Departmental Library, University Medical Center Mainz, Johannes Gutenberg University Mainz, Mainz, Germany

**Keywords:** vulvar cancer, PD-L1, PD-1, pembrolizumab, immune checkpoint inhibitor, meta-analysis

## Abstract

**Introduction:**

Vulvar cancer carries a favourable prognosis in early stages. However, therapeutic options for advanced or recurrent cases are limited despite a variety of therapeutic modalities, such as extensive surgical resection, chemotherapy, and radiotherapy. The most important emerging treatment modalities are immune checkpoint inhibitors. This systematic review and meta-analysis aims to assess the efficacy and safety of pembrolizumab, an immune checkpoint inhibitor, in women with advanced vulvar cancer.

**Materials and methods:**

Following a comprehensive search, review, and appraisal, two relevant single-arm studies were included. Meta-analysis was conducted using R4.3.0 software and RStudio 2023.03.0, presenting the overall effect size with a 95% confidence interval. Heterogeneity was assessed using I^2^ and the Cochrane Q χ2 statistics.

**Results:**

Out of 154 studies screened for eligibility, two single-arm studies involving 119 patients receiving pembrolizumab for advanced vulvar cancer were included. The pooled objective response rate (ORR) was overall 10% (95% CI: 0.00-0.84) and 9% (95% CI: 0.00-0.89) in the PD-L1 positive subgroup. In the intention-to-treat (ITT) population, 31% (95% CI: 0.04-0.85) exhibited any clinical benefit (complete response, partial response, or stable disease). In the ITT population at six months, progression-free survival (PFS) was 19% (95% CI: 0.01-0.82), and overall survival (OS) was 48% (95% CI: 0.08-0.90). At 12 months, PFS decreased to 9% (95% CI: 0.00-0.85), and OS was 33% (95% CI: 0.04-0.85). No statistically significant heterogeneity was observed in PFS and OS analyses.

**Discussion and conclusion:**

This study suggests that one-third of women with advanced or recurrent vulvar cancer may, without the influence of PD-L1 status, benefit from pembrolizumab treatment despite a decline in both PFS and OS at 12 months. These findings provide support for considering pembrolizumab in the treatment paradigm for this specific subset of cancer patients.

**Systematic review registration:**

https://www.crd.york.ac.uk/prospero/, identifier CRD42023391888

## Introduction

Vulvar cancer, which accounted for 45240 cases and resulted in 17427 fatalities worldwide in 2020, represents approximately 5% of all gynecological malignancies (1). The most predominant histological subtype is squamous carcinoma (SCC). The pathogenesis of vulvar cancer can be delineated into two principal oncogenic pathways: the human papillomavirus (HPV)-dependent and HPV-independent pathways. Recent meta-analysis data revealed a 39.1% prevalence of high-risk HPV in vulvar cancer cases (2). Notably, the incidence of HPV-dependent vulvar cancer is higher in younger women, while the HPV-independent pathway, often linked to preexisting chronic vulvar conditions like lichen sclerosus, is more frequently observed in the elderly population (3).

In women with early-stage vulvar cancer, the gold standard of treatment is surgical excision of the tumor, followed, when appropriate, by radio-chemotherapy administered in accordance with established national and international guidelines (4, 5). Recurrence is observed in up to 24% of these patients following initial treatment (4). In cases of early-stage disease, the five-year survival rate is high, reaching up to 90%. Conversely, for women facing recurrent vulvar cancer, the five-year survival rates are considerably low: 50%-70% in cases of local recurrence, up to 27% in lymphatic recurrence, and up to 14% in instances of distant recurrence (6).

Dealing with women suffering from advanced, recurrent, metastatic, or heavily pretreated cancer of the vulva, the array of available treatment modalities – ranging from radical surgical interventions to radiotherapy and systemic therapies – is often limited. This limitation may be attributed to the tumor´s extent, the patient´s frailty, or previous unsuccessful therapeutic approaches. Furthermore, it is essential to underline the scarcity of the data available concerning managing advanced and recurrent cancer of the vulva, primarily due to the small number of cases documented in published literature, which precludes the establishment of a standardized care protocol (4). As a result, clinicians often deduce therapeutic approaches for women with vulvar cancer by extrapolation from the treatment options of patients with cervical, anal, or head and neck cancers (4). In cases with advanced, recurrent, metastatic, and/or heavily pretreated vulvar cancer, a “best supportive care” strategy is also considered suitable (4). Nevertheless, emerging therapeutic possibilities, exemplified by immune checkpoint inhibitors, are becoming increasingly promising, offering additional therapeutic options.

The immune response of humans is intricately regulated through checkpoint pathways. Tumor cells skilfully exploit these pathways to evade the detection and destruction by the immune system (7). Several factors come into play in shaping the naturally occurring anti-tumor T cell response, including inadequate tumor antigenicity, intrinsic interferon-γ signaling, downregulation of major histocompatibility complex (MHC) expression, and the orchestration of oncogenic signaling (8).

Several mechanisms and biomarkers indicative of tumor-intrinsic resistance have been identified to date. These include the expression of immune checkpoint molecules, such as PD-L1 expression in tumor cells and tumor-infiltrating lymphocytes, high tumor mutational burden (TMB-H), mismatch repair deficiency (dMMR), and high level of microsatellite instability (MSI-H) (7, 8). Immune checkpoint inhibitors, like anti-PD-1 or anti-PD-L1 agents, play a pivotal role in surmounting tumor-intrinsic resistance. Evidence from the KEYNOTE-028 basket trial underscores the anti-tumor effects of pembrolizumab in diverse PD-L1 positive, advanced, solid tumors, yielding an objective response rate (ORR) spanning from 30% in esophageal cancer to 0% in pancreatic cancer (9).

The Food and Drug Administration (FDA) has approved pembrolizumab in any cancer with PD-L1 positivity, TMB-H, dMMR, or MSI-H (4). Consequently, the current National Comprehensive Cancer Network (NCCN) guidelines acknowledge pembrolizumab as second-line therapy in advanced recurrent and metastatic disease of the vulva displaying the aforementioned biomarker profile (4). The European Society of Gynecological Oncology (ESGO) Guidelines for the management of patients with vulvar cancer suggest that the addition of pembrolizumab may be considered for selected patients with metastatic or recurrent unresectable disease (10). It is important to note that, up to now, the European Medicines Agency (EMA) has not approved checkpoint inhibitors for the treatment of women with vulvar cancer (11).

## Aim of the study

The aim of the study was to assess the efficacy of pembrolizumab in women with advanced vulvar cancer, primarily in terms of survival rates. We performed a systematic review and a meta-analysis.

## Methods

This systematic review and meta-analysis follows the Preferred Reporting Items for Systematic Reviews and Meta-Analyses (PRISMA) statement (12). The research was registered prospectively in PROSPERO by the ID CRD42023391888.

### Search strategy

A comprehensive search syntax using MESH and free text terms for vulvar cancer and treatment with the immune checkpoint pembrolizumab was developed by a medical librarian (LCO) in consultation with a topic expert (RS). The strategy was developed for MEDLINE (via PubMed). It was adapted appropriately for the Cochrane Central Register of Controlled Trials (via The Cochrane Library) and Web of Science Core Collection (via Web of Science). All databases were searched from inception to February 6^th^, 2023.

Additionally, ClinicalTrials.gov was searched to identify ongoing trials. We also explored the grey literature on Google Scholar. The first 100 results were selected and screened.

The main keywords for the literature search were: Anti PD L1, Anti PD 1, MK-3475, Keytruda, SCH-900475, Pembrolizumab, vulvar malignancy, vulvar neoplasm, vulvar carcinoma, vulvar cancer.

The reproducible searches for all databases are available in the **Online Supplementary**.

### PICO criteria (population, intervention, control, outcomes)

Population: women with advanced, recurrent or metastatic vulvar cancer.

Intervention: treatment with pembrolizumab.

Control: no treatment with pembrolizumab.

Primary outcomes: Efficacy outcomes, including progression-free survival (PFS), and overall survival (OS) and objective response rate (ORR).

Secondary outcomes: complete response (CR), partial response (PR), stable disease (SD), progressive disease (PD), any benefit (defined as the subpopulation with SD, CR, and PR), and adverse events.

### Inclusion and exclusion criteria of the included studies

The inclusion criteria were: prospective clinical trials (randomized controlled trials and observational studies), women treated with pembrolizumab for advanced, persistent, recurrent or metastatic vulvar cancer.

The exclusion criteria were: articles regarding *in-vitro* experiments, pathological studies, conference papers, opinion articles, or editorials. The most recent study was included if authors published several articles using the same data set.

### Quality assessment of the included studies

The study quality assessment was conducted using the Methodological Evaluation Metrics for Non-Randomized Controlled Trials (MINORS), a tool validated for the appraisal of single-arm studies (13). Two independent investigators (RS and LJS) performed the quality assessment, and disagreement was resolved by discussion. MINORS encompass a set of 12 evaluation indicators, with each indicator being assigned a score of 0 to 2 (0 = absence of reported data, 1 = data reported but lacking sufficient information, 2 = data reporting accompanied by adequate and comprehensive information). he first eight indicators apply to studies conducted without a control group, with a maximum score of 16.

### Data extraction

Two independent investigators (RS and LJS) performed the study selection, and the discussion resolved disagreement.

The included studies’ characteristics were recorded: authors, year of publication or report results, study type, sample size, therapeutic regimen, follow-up period, disease status, number of patients, reported endpoints, and criteria for response.

Efficacy outcomes, including ORR, CR, PR, SD, PD, any benefit, PFS, and OS, were recorded in self-designed original data sheets. We had no access to the original survival data. We extracted the data and the number censored from Kaplan-Meier (K-M) curves, the number at risk published under the K-M curves, and the number of events.

For the endpoints PFS, OS, CR, PR, SD, PD, any benefit, the efficacy outcomes were assessed using, on one hand, the intention to treat (ITT) population, which means the registered events were calculated regarding the total number of study participants, on the other hand by taking in count the population, which was assessed by per-protocol analysis (PPA). In the PPA group, the total number of participants was calculated by subtracting those patients from the total number of participants who discontinued the study or were lost to follow-up for the particular time points, and no assessment of the particular study endpoints was obtained.

The data were extracted by RS and LJS.

### Statistical analysis

The R4.3.0 software and RStudio 2023.03.0 with the metafor, meta, and tidyverse packages were employed to conduct the meta-analysis. The random-effect model was used to account for the heterogeneity of the studies, for differences between the included studies, to provide a more general estimate of the overall effect, and to enable a more realistic and flexible approach to the mentioned inherent variability of the studies. The random-effect model assumes, that the effects observed across the included studies follow a certain distribution, typically assumed to be normal (14). As the participant sample of the included studies might vary over several characteristics, we considered the fixed-effect model unsuitable for this study. The overall effect size estimated from the combination of the retrieved studies included in the meta-analysis was presented as a point estimate with a 95% confidence interval.

The heterogeneity (degree of variation) between the studies included in the analysis indicated the variability or differences in the effect size or outcomes. It was quantified by using both I^2^ and the Cochrane Q χ^2^ statistics. Significant heterogeneity was assumed for p-value < 0.05 in the Q-test and a value greater than 50% in the I^2^ statistics. According to Cochrane’s handbook, the heterogeneity of the I^2^ statistics of 0% to 40% might not be important, the I^2^ of 30% to 60% may represent moderate heterogeneity, an I^2^ of 50% to 90% may represent substantial heterogeneity and results of I^2^ of 75% to 100% may represent considerable heterogeneity (14).

Baujat plots were employed to investigate the source of heterogeneity visually. Publication biases were analyzed by graphical analysis using funnel plots.

### Patient and public involvement

No patients were involved in planning the research question, design, study implementation, or results interpretation.

## Results

**Figure 1:**
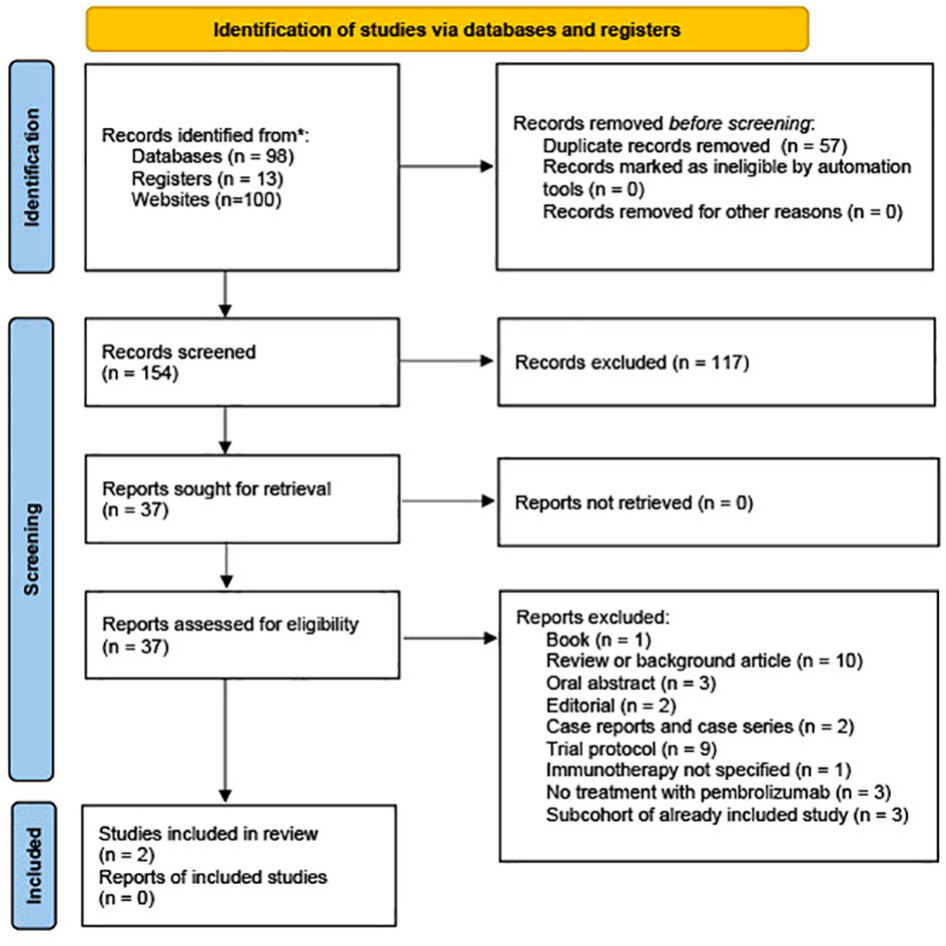
PRISMA flow chart.

The initial search conducted in PubMed (n = 50), the Cochrane Library (n = 4), ClinicalTrials.gov (n = 13), Web of Science (n=44), and Google Scholar (n=100) yielded a total of 211 relevant references. Following the import of these references into EndNote 20.0.1, an automated duplication process was executed using Deduklick, an AI-based deduplicate solution (15). This process led to removing duplicate records, resulting in 154 unique articles. Of the remaining 37 articles subjected to full-text screening, 35 were excluded for various reasons. A visual representation of the study selection process is presented in **Figure 1**. One publication by How et al. was excluded because it reported a case series originating from a basket trial. This case series encompassed a patient with vulvar cancer who was included in the study cohort for vaginal cancer, and it was noted that this patient exhibited the most significant tumor load in the vagina. The authors of the study acknowledged the ambiguity regarding whether the tumor was linked to recurrent vulvar cancer or represented *de novo* vaginal cancer (16).

Only two reports met the systematic review and meta-analysis inclusion criteria and reported treatment of women with advanced or metastasized vulvar cancer with pembrolizumab (9, 17).

The collective data from these studies included a total of 119 women diagnosed with vulvar cancer, of whom 102 women tested positive for PD-L1 expression.

### Characteristics of the studies

The characteristics of the included articles are presented in **Table 1**.

**Table 1:** Characteristics of the included studies.

	KEYNOTE-028 (9)	KEYNOTE-158 (17)
Study identifier (unique trial registration number)	NCT02054806	NCT02628067
Study type	Non-randomized, multicentre, cohort phase Ib, single-arm, open-label	Non-randomized, multicentre, multicohort, phase 2, single-arm, open-label
Primary inclusion criteria	advanced (unresectable and metastatic) vulvar SCC	advanced (metastatic and unresectable) vulvar SCC
Number of patients included	18	101
PD-L1 status by a combined positive score	PD-L1 positive	PD-L1 positive (n=84), unknown (n=10), and negative (n=7)
Intervention	Pembrolizumab 10mg/kg q2w i.v. for a maximum of 2 years	Pembrolizumab 200 mg i.v. q3w up to 35 cycles or two years
Controls	none	none
Primary endpoints	ORR per RECIST v1.1	ORR per RECIST v1.1
Secondary endpoints	PFS, OS, AE	PFS, OS, AE
Median time to data cut-off in months for vulvar SCC	n.s.	36.9
Duration of response in months in women with vulvar SCC	n.s.	20.4
Stage IV disease in % in women with vulvar SCC	n.s.	88.1
PD-L1 positivity in % in women with vulvar SCC	100	83.2
Median age in women with vulvar SCC	n.s.	64
Chemotherapy (2 lines) before pembrolizumab treatment in women with vulvar SCC in % of women with vulvar SCC	n.s.	33.7
Radiation before pembrolizumab treatment in women with vulvar SCC in % of women with vulvar SCC	n.s.	92.1
ECOG in women with vulvar SCC	n.s.	0,1

PD-L1, programmed death ligand 1; SCC, squamous cell cancer; RECIST, Response Evaluation Criteria In Solid Tumors; ORR, objective response rate; PFS, progression-free survival; OS, 0verall survival; AE, adverse events; ECOG, Eastern European Cooperative Oncology Group; n.s., not specified.

### Quality assessment and publication bias of the studies

Publication bias was evaluated using Funnel (**Supplementary Material**) for all outcomes under investigation. The funnel plots exhibited a general symmetrical distribution, suggesting a relatively balanced representation of published studies.

The methodological quality and validity of the included studies were appraised utilizing the Methodological Index for Non-Randomized Studies (MINORS) scale (13). The cumulative score for KEYNOTE-028 was 14, while for KEYNOTE-158, it amounted to 15 (**Table 2**) (out of a maximum cumulative score of 16). These scores indicate a good quality of both studies included in this analysis. The specific rationale behind the specific MINORS sub-scores for each study assessed was included into the **Supplementary Material**.

**Table 2:** MINORS risk of bias assessment.

MINORS criteria	KEYNOTE-028, MINORS points	KEYNOTE-158, MINORS points
1 A clearly stated aim: The question addressed should be precise and relevant in the light of available literature	2	2
2 Inclusion of consecutive patients: all patients potentially fit for inclusion (satisfying the criteria for inclusion) have been included in the study during the study period (no exclusion or details about the reasons for exclusion)	1	1
3 Prospective collection of data: data were collected according to a protocol established before the beginning of the stud	2	2
4 Endpoints appropriate to the aim of the study: unambiguous explanation of the criteria used to evaluate the main outcome, which should be in accordance with the question addressed by the study. Also, the endpoints should be assessed on an intention-to-treat basis.	2	2
5 Unbiased assessment of the study endpoints: blind evaluation of objective endpoints and double-blind evaluation of subjective endpoints. Otherwise, the reasons for not blinding should be state	1	2
6 Follow-up period appropriate to the aim of the study: the follow-up should be sufficiently long to allow the assessment of the main endpoint and possible adverse events	2	2
7 Loss to follow-up less than 5%: all patients should be included in the follow-up. Otherwise, the proportion lost to follow-up should not exceed the proportion experiencing the major endpoint	2	2
8 Prospective calculation of the study size: information on the size of detectable difference of interest with a calculation of 95% confidence interval, according to the expected incidence of the outcome event, and information about the level for statistical significance and estimates of power when comparing the outcomes	2	2
Cumulative score	14	15

### Objective response rate

**Figure 2:**
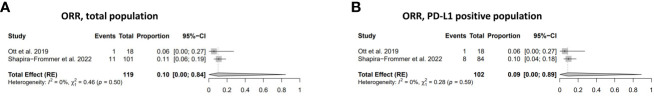
ORR in advanced vulvar cancer. **(A)** total population. **(B)** PD-L1 positive population.

The combined effect size, estimating the overall ORR across the studies for individuals with PD-L1 positive, unknown, or negative status, was calculated to be 0.10 (95% CI: 0.00; 0.84). The ORR within the PD-L1 positive study population was found to be 0.09 (95% CI: 0.00; 0.89) (**Figure 2**). The I^2^ and χ^2^ statistics, which are indicators of heterogeneity, suggested minimal to negligible heterogeneity across the studies in both of the analyses.

### Survival

#### Progression-free survival

**Figure 3:**
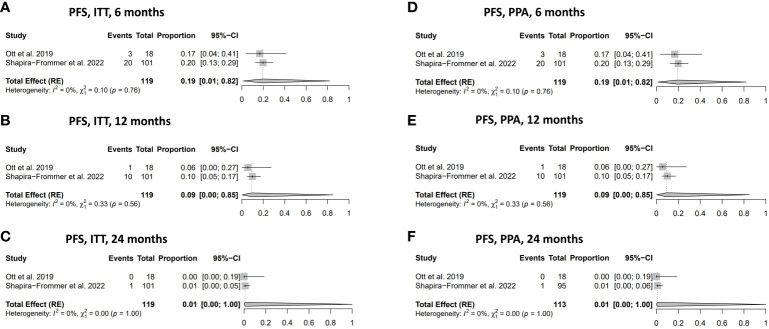
Progression-free survival (PFS). **(A)** PFS in the ITT population at 6 months. **(B)** PFS in ITT population at 12 months. **(C)** PFS in ITT population at 24 months. **(D)** PFS in PPA population at 6 months. **(E)** PFS in PPA population at 12 months. **(F)** PFS in PPA population at 24 months. ITT, intention to treat; PPA, per protocol analysis.

The PFS rates in women diagnosed with vulvar cancer in both the ITT and the PPA populations were: 19% (95% CI: 0.01-0.82) at six months, 9% (95% CI: 0.00-0.85) at 12 months, respectively (**Figures 3A, B, D, E**). The PFS at 24 months in the ITT and the PPA populations was 1% (95% CI 0.01-1.00) at 24 months in both groups (**Figures 3C, F**). No statistically significant heterogeneity was observed in relation to the PFS analyses.

#### Overall survival

**Figure 4:**
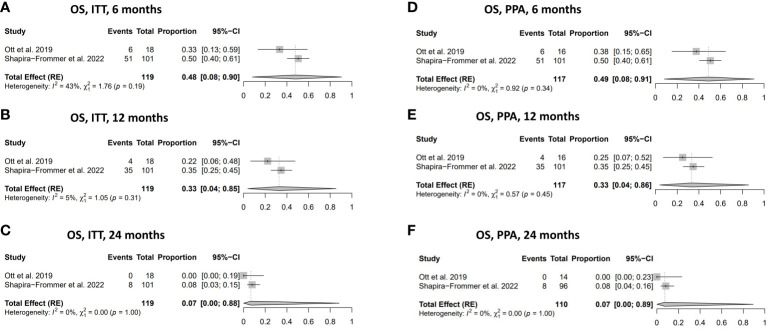
Overall survival (OS). **(A)** OS in ITT population at 6 months. **(B)** OS in ITT population at 12 months. **(C)** OS in ITT population at 24 months. **(D)** OS in the PPA population at 6 months. **(E)** OS in PPA population at 12 months. **(F)** OS in PPA population at 24 months. ITT, intention to treat; PPA, per protocol analysis.

The overall survival in women with vulvar cancer who received treatment with pembrolizumab demonstrated favourable outcomes, with a 49% (95% CI: 0.08-0.91) OS rate at six months in the PPA population and 48% (95% CI: 0.08-0.90) in the ITT population (**Figures 4A, D**). The OS rate gradually decreased to 33% at 12 months in both the ITT and the PPA populations and substantially declined to 7% at 24 months in the ITT and PPA populations (**Figures 4B, C, E, F**). No statistically significant heterogeneity was observed in the analysis of OS (**Figures 4A–F**).

### Tumor response

**Figure 5:**
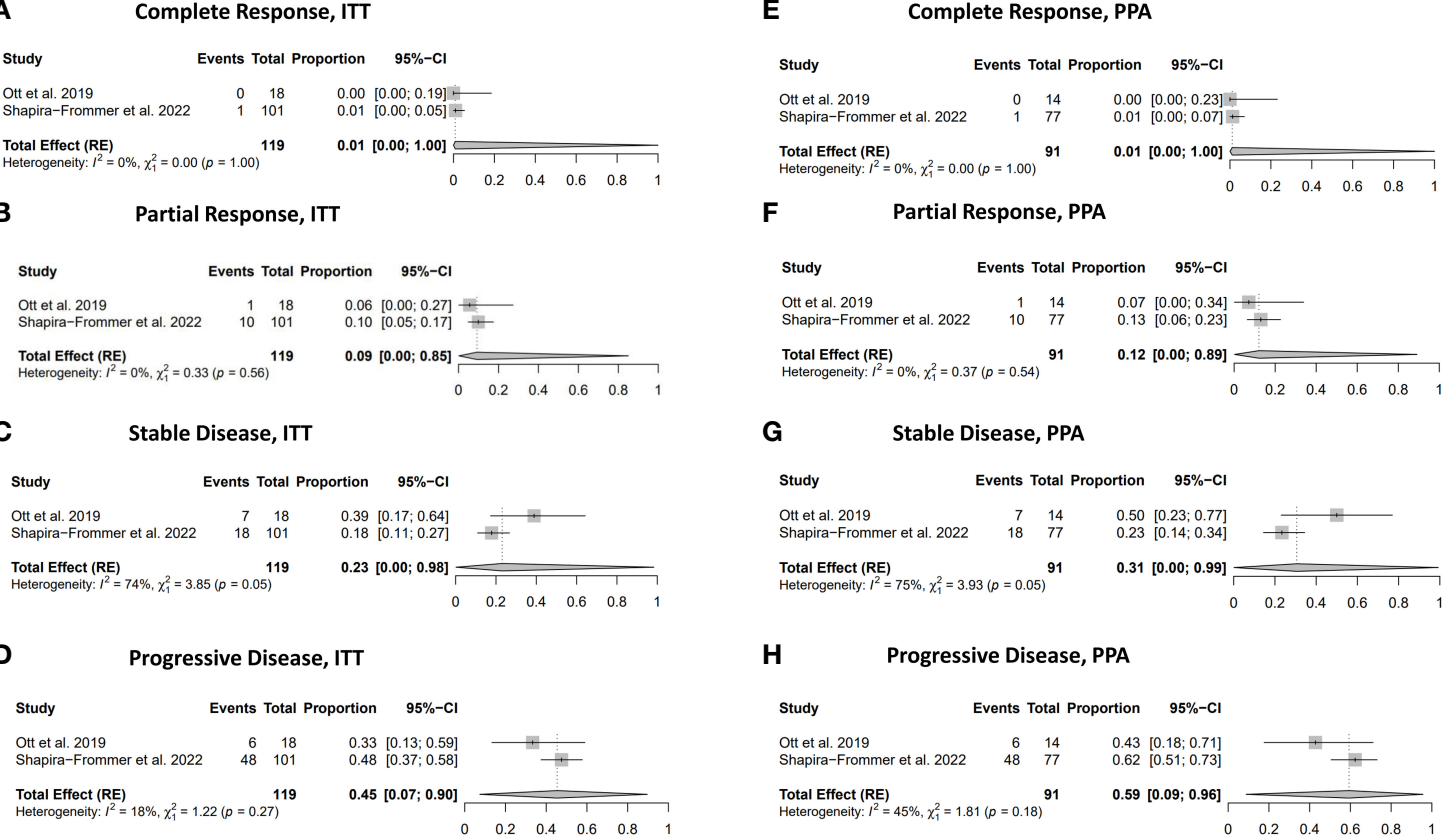
Response rate. **(A)** Complete response in ITT population. **(B)** Partial response in ITT population. **(C)** Stable disease in ITT population. **(D)** Progressive disease in PPA population. **(E)** Complete response in PPA population. **(F)** Partial response in PPA population. **(G)** Stable disease in PPA population. **(H)** Progressive disease in PPA population.

Only 1% of the ITT and the PPA population achieved complete response after treatment with pembrolizumab. Still, the results did not reach statistical significance (**Figures 5A, E**). Partial response was observed in 9% of the ITT population and 12% of the PPA population (**Figures 5B, F**). In comparison, stable disease was achieved in 23% of cases of the ITT population and 31% of the PPA population (**Figures 5C, G**). Progressive disease was observed in 45% of cases in the ITT population and 59% in the PPA population (**Figures 5D, H**). Substantial heterogeneity was detected in the results dealing with stable disease in both the ITT population [I^2^ = 74% and χ^2^ = 3.85 (p=0.05)] and the PPA population [I^2^ = 75% and χ^2^ = 3.93 (p=0.05)]. In contrast, no substantial heterogeneity was detected in the other analyses (CR, PR, and PD) dealing with the tumor response to pembrolizumab.

In total, a proportion of 31% (95% CI: 0.04-0.85) showed any clinical benefit (CR, PR and SD) in the ITT population and 41% (95% CI: 0.04-0.91) in the PPA population after treatment with pembrolizumab. Some degree of heterogeneity was seen in the outcomes of any benefit in the ITT population [I^2^ = 42% and χ^2^ = 1.72 (p=0.19)] and in the PPA population population [I^2^ = 45% and χ^2^ = 1.81 (p=0.81)].

## Discussion

This study represents the first meta-analysis assessing the efficacy of pembrolizumab in the context of advanced, recurrent, or metastatic vulvar cancer.

Within a cohort of 119 women, including those with PD-L1 positive, unknown, or negative tumors, we observed an ORR of 10% (95% CI 0.00-0.84). Among the 102 women who tested positive for the PD-L1 biomarker before starting pembrolizumab treatment, the ORR was 9% (95% CI 0.00-0.89). The KEYNOTE-028 study reported a relatively low ORR of 6% in 18 patients following pembrolizumab treatment despite the entire study cohort displaying PD-L1 positivity (9). Similarly, the KEYNOTE-158 study reported a low ORR in the total group of study participants and the PD-L1 positive subgroup (17). The authors of the KEYNOTE-158 study did not specify the involvement of other mechanisms contributing to immune evasion, such as dMMR/MSI-H, in the PD-L1 negative subgroup of vulvar cancer patients (17).

In contrast, other cancers characterized by squamous subtype and HPV-positivity exhibited more favourable response rates. For instance, anal canal SCC displayed an ORR of 18%, oesophageal SCC/adenocarcinoma showed an ORR of 30%, and cervical SCC exhibited an ORR of 17% (9). Nivolumab, another immune checkpoint inhibitor, showed an ORR of 20% in women with vaginal/vulvar cancer, albeit with only five women with vulvar cancer included in the CeckMate 358 trial (18). Due to the limited number of vulvar cancer patients treated with nivolumab, it remains challenging to draw definitive conclusions regarding the comparative suitability of pembrolizumab versus nivolumab in improving outcomes of women with advanced, metastatic, or recurrent vulvar cancer.

In HPV-related cancers, the overexpression of PD-1 on T-cells and PD-L1 on myeloid cells and tumor cells was frequently detected (19, 20). PD-L1 was expressed in up to 51% of cervical tumor cells, which are primarily high-risk HPV-positive (20). In patients with vulva cancer, PD-L1 expression was identified in up to 32%-43% of cases in cancer cells and up to 60.7%-81% of patients in peritumoral immune cells (21, 22). Furthermore, in metastatic tumors of the vulva, PD-L1 positivity was documented in up to 50% of cases (22). This suggests that over half of vulvar cancer patients may meet the criteria for therapy with pembrolizumab as per NCCN guidelines (4). Moreover, PD-L1 positivity was associated with poorer clinical outcomes, particularly in a subset of patients with high-risk HPV-negative tumors (23). Therefore, pembrolizumab therapy may hold promise in improving the prognosis of women at higher risk for adverse outcomes.

However, it is not only the presence but also the pattern of PD-L1 expression that appears to influence the survival of patients with HPV-related cancers. Women with cervical SCC who displayed diffuse PD-L1 expression evolved worse in terms of survival compared to those with tumor-stroma margin PD-L1 expression (24). In vulvar cancer, one study revealed that PD-L1 positivity of peritumoral immune cells independently correlated with a favourable OS outcome (21). In the studies included in this meta-analysis, PD-L1 status was assessed using a combined positive score (CPS), which considers the number of PD-L1 staining cells and the total number of viable tumor cells (9, 17). Consequently, this meta-analysis does not allow us to conclude whether a specific pattern of PD-L1 expression predicts a better response to the therapy with pembrolizumab and, subsequently, improved survival.

Our meta-analysis elucidates that PD-L1 expression alone does not necessarily indicate a positive response to pembrolizumab and that PD-L1-negative vulvar cancer patients may also benefit from this treatment. Similar phenomena have been documented in other cancer types. Anti-tumor responses were not solely determined by PD-L1 expression, as previously observed in melanoma and non-small lung cancer (25–27). They are also influenced by MSI-H/dMMR, tumor mutational burden, and potentially other biomarkers (25–28).

Moreover, our meta-analysis showed an ORR of 10% in the entire population but only 9% in the PD-L1 positive subgroup. This implies that PD-L1 negative and unknown participants of the KEYNOTE-158 study exhibited equal responses compared to those who expressed the PD-L1 biomarker (17). This suggests that factors other than the PD-L1 expression may be better suitable for predicting pembrolizumab response in women with vulvar cancer and that PD-L1 negative patients may also benefit from this therapy, even though it has not yet received official approval. Variability in the predictive accuracy of PD-L1 expression regarding the responses to immune checkpoint inhibitors has been documented across multiple oncological contexts, notably in cases of triple-negative breast cancer and in cervical cancer patients undergoing concurrent radiochemotherapy and immunotherapy regimens (29, 30). This heterogeneity in response prediction underscores the complexity inherent in the interaction between oncological treatments and immune modulation, necessitating further elucidation in diverse cancer subtypes.

The survival outcomes presented in this study showed a favourable response at six months with a 48% overall survival rate in the ITT population, a slight decline at 12 months with a 33% survival rate, and a rapid decrease at 24 months with a 7% overall survival rate. The PFS in both the ITT and PPA populations exhibited lower rates at 6, 12, and 24 months, with 19%, 9%, and 1%, respectively. Women with vulvar cancer appear to respond less favourable to pembrolizumab treatment than women with cervical cancer (31, 32), for whom the OS was up to 54.4% and the PFS approximately 30% at 24 months (31). This discrepancy suggests that additional pathogenetic and molecular factors influencing immune evasion may negatively affect the prognosis of vulvar cancer patients. In neoplasms characterized by chronic inflammation, such as vulvar cancers in women with a history of lichen sclerosus, there is a predilection for the dominance of negative immune regulatory factors. This dominance can potentially attenuate the therapeutic efficacy of immune checkpoint inhibitors, and the presence of an established immunosuppressive tumor microenvironment may significantly diminish the effectiveness of immunotherapeutic interventions (33).

The expression of the HPV-E7 oncoprotein has been associated with increased PD-L1 expression of the intra-tumoral surface and worse prognosis in women with cervical cancer (34). Additionally, the HPV-related E5 protein has been described to hinder the presentation of non-viral associated antigens on MHC molecules and the activation of anti-tumor T cells, potentially leading to resistance to immune checkpoint blockade and poorer survival in head and neck cancer patients (35, 36). However, this effect was effectively counteracted by rimantadine, an E5 protein inhibitor (36). The expression of E5 and E7 might represent one mechanism contributing to resistance to immune checkpoint inhibitor therapy in HPV-positive women with vulvar cancer, and a combination of E5 inhibitor rimantadine with pembrolizumab may improve the effectiveness of the immune checkpoint inhibition in the HPV-positive subgroup of females with vulvar or cervical cancers.

Further studies are warranted to identify potential treatment strategies that could improve the response to immune checkpoint inhibitors among women with advanced vulvar cancer. Chemotherapy has emerged as an additional promising therapeutic modality, given its capacity to catalyze a tumor-specific immune response. This phenomenon has been notably observed in non-small-cell lung cancer with chemotherapy inducing immunogenic cell death and facilitating the release of neoantigens to be recognized by antigen-presenting cells (37).

In support of this notion, a recent meta-analysis examining the efficacy of PD-1/PD-L1 inhibitors in ovarian cancer unveiled outcomes similar to those found in the present study – namely, a low ORR (38). However, a significant improvement in ORR, reaching 36%, was reported in ovarian cancer patients subjected to a combined treatment regimen involving chemotherapy and PD-1/PD-L1 inhibitors (38). Additionally, the combination of durvalumab and bevacizumab alongside systemic chemotherapy revealed a statistically significant and clinically meaningful improvement in PFS in women with ovarian cancer (39). This underscores the potential of chemotherapy to increase the efficacy of immune checkpoint inhibitors in specific cancer types. An additional option is the concurrent administration of vascular endothelial growth factor (VEGF) inhibitors alongside immune checkpoint inhibitors. Existing data indicate a synergistic effect between antiangiogenic agents and PD-1/PD-L1 inhibitors in solid tumors, including conditions like endometrial cancer, non-small-cell lung cancer, and renal cancer. Notably, vulvar cancer exhibits a moderate to strong VEGF expression in up to 13.9% of cases (40). In ovarian cancer, the addition of anti-VGEF agents led to a notable increase in ORR, elevating it from 9% in immune checkpoint monotherapy to 30% when combined with anti-VEGF treatment (38). Moreover, maintenance therapy with olaparib, pembrolizumab, and bevacizumab showed durable efficacy in a subset of ovarian cancer patients (41). Consequently, drawing an analogy with ovarian cancer, the therapy with immune checkpoint inhibitors combined with bevacizumab, e.g. as maintenance therapy, may improve prognosis and may expand the therapeutic options in women with vulvar cancers.

Another promising therapeutic option, which already showed antitumor activity in advanced cervical cancer, is the combination of bevacizumab, pembrolizumab, or chemotherapy and tisotumab vedotin (a tissue factor-directed antibody-drug conjugate) (42). This combination therapy may also improve the response rate and the duration of response of immune checkpoint inhibitors in second-line treatment in women with advanced vulvar cancer, but additional clinical trials with this tumor entity are warranted.

Furthermore, the potential for increasing treatment response may be explored by combining two checkpoint inhibitors acting on different tumor-intrinsic resistance mechanisms. This approach has demonstrated promise in improving melanoma patients’ response rates compared to monotherapy treatment (26). Consequently, combination therapy might also optimize outcomes for women with vulvar cancer, potentially increasing the modest response rates observed in monotherapy employing pembrolizumab while extending the durability of the response through a second checkpoint inhibitor.

Another strategy might be the local administration of low-dose immune checkpoint inhibitors. This approach aims to harness increased immunity by modulating the immune response within the primary tumor site and the drained lymph nodes (43). It presents a potential strategy to be explored in the future, offering additional therapeutic options.

## Strengths and limitations

This study is the first meta-analysis exploring the impact of pembrolizumab on women with advanced cancer of the vulva. We analyzed both the ITT and the PPA populations with no significantly different results. This underscores the robustness of the findings.

However, it is imperative to view the findings of this study within the context of certain limitations. Publication bias was assessed by funnel plots. Nevertheless, as less than 10 studies were included, the power of funnel plots may be too low to distinguish chance from real asymmetry. It cannot be disregarded that negative outcomes, possessing the potential to significantly alter the conclusions of this pooled analysis, remained unpublished. Next, the absence of randomized controlled trials is a noteworthy constraint, as this analysis relies on observational-single-arm studies. Owing to the scarcity of available studies, only a relatively small cohort of 119 women with advanced vulvar cancer could be incorporated in this meta-analysis. This limitation inherently restricts the extrapolative applicability of our findings, as the results based on a small specific group may not be accurately applicable to a larger and more diverse group. The rarity of the underlying condition and the small study size limit the generalizability in a wider population.

Furthermore, notable heterogeneity, ranging from moderate to substantial, was observed in some of the outcome measures. These variations may be attributed to differences in the demographic characteristics of the study populations, including factors such as age, Eastern Cooperative Oncology Groupe (ECOG) status, and PD-L1 status. As only two studies were included, we were unable to specify the source of heterogeneity using meta-regression and subgroup analysis. The examination of response duration, mean PFS and mean OS was precluded, as the raw data from the included studies was not accessible. Moreover, a pooled meta-analysis regarding the adverse effects of pembrolizumab, specifically in women with vulvar cancer, was not performed, as only one study specified the adverse effects in this specific collective of patients.

## Conclusion

In conclusion, this single-arm meta-analysis suggests that pembrolizumab therapy may elicit a tumor response and potentially contribute to prolonged survival in patients with advanced, recurrent or metastatic vulvar cancer, especially when administered as second or later-line treatment. The therapeutic efficacy of pembrolizumab may be more pronounced particularly when administered in combination with a second immune checkpoint inhibitor or with chemotherapy, offering a valuable treatment modality for individuals with limited alternative therapeutic options. The findings of this study may bolster the consideration of pembrolizumab treatment for this subset of cancer patients. The results support the current therapeutic approaches advocated by organizations such as NCCN and ESGO. Additional data, ideally derived from randomized controlled trials with larger sample sizes, including tumors with and without PD-L1 expression, are crucial. Furthermore, studies focused on assessing the efficacy of immune checkpoint inhibitors during adjuvant therapy are essential to validate the effectiveness of this treatment modality in patients with vulvar cancer. This further research is pivotal in confirming the role of pembrolizumab and similar agents in the therapeutic landscape of vulvar cancer. These additional investigations are essential for expanding the therapeutic arsenal available for this rare form of cancer. The outcomes of such research could significantly enhance our understanding and management of vulvar cancer, potentially leading to improved patient outcomes.

## Data availability statement

The original contributions presented in the study are included in the article/**Supplementary Material**. Further inquiries can be directed to the corresponding author.

## Author contributions

RS: Conceptualization, Data curation, Formal analysis, Investigation, Methodology, Project administration, Supervision, Validation, Visualization, Writing – original draft, Writing – review & editing. LS: Conceptualization, Investigation, Methodology, Visualization, Writing – original draft, Writing – review & editing. LC: Conceptualization, Methodology, Writing – review & editing. PK: Conceptualization, Visualization, Writing – review & editing. MWS: Methodology, Visualization, Writing – review & editing. KA: Visualization, Writing – review & editing. AH: Visualization, Writing – review & editing. WB: Writing – review & editing. KS: Visualization, Writing – review & editing. MS: Visualization, Writing – review & editing. AH: Visualization, Writing – review & editing, Investigation.
