# Supplementary material for: Efficacy of pembrolizumab in advanced cancer of the vulva: a systematic review and single-arm meta-analysis
Source: Front Oncol. 2024 Feb 19;14:1352975. doi: 10.3389/fonc.2024.1352975 (PMC10910062; doi:10.3389/fonc.2024.1352975)
Supplement: Supplementary file 1 [file DataSheet_1.docx]

# Supplement materials

# Search strategies

**Table S1.** Search strategy for MEDLINE via PubMed.

| **Search number** | **Query** | **Results** |
| --- | --- | --- |
| #3 | #1 AND #2 | 50 |
| #2 | ("humanized antibodies"[Title/Abstract] OR "programmed cell death protein 1 inhibit*"[Title/Abstract] OR "pd 1 inhibit*"[Title/Abstract] OR "cytotoxic t lymphocyte associated protein 4 inhibitor*"[Title/Abstract] OR "ctla 4 inhibitor*"[Title/Abstract] OR "pd 1 pd l1 block*"[Title/Abstract] OR "programmed death ligand 1 inhibit*"[Title/Abstract] OR "pd l1 inhibit*"[Title/Abstract] OR "immune checkpoint block*"[Title/Abstract] OR "immune checkpoint inhibit*"[Title/Abstract] OR "Anti-PD-L1"[Title/Abstract] OR "Anti-PD-1"[Title/Abstract] OR "Lambrolizumab"[Title/Abstract] OR "MK-3475"[Title/Abstract] OR "Keytruda"[Title/Abstract] OR "SCH-900475"[Title/Abstract] OR "Pembrolizumab"[Title/Abstract] OR "Pembrolizumab"[Supplementary Concept] OR "Immune Checkpoint Inhibitors"[Pharmacological Action] OR "Immune Checkpoint Inhibitors"[MeSH Terms] OR "antibodies, monoclonal, humanized"[MeSH Terms:noexp]) | 92.237 |
| #1 | "vulva intraepithelial neoplas*"[Title/Abstract] OR "vulvar intraepithelial neoplas*"[Title/Abstract] OR "vulval intraepithelial neoplas*"[Title/Abstract] OR "vSCC"[Title/Abstract] OR "vulva malignan*"[Title/Abstract] OR "vulvar malignan*"[Title/Abstract] OR "vulva carcinom*"[Title/Abstract] OR "vulvar carcinom*"[Title/Abstract] OR "vulva neoplas*"[Title/Abstract] OR "vulvar neoplas*"[Title/Abstract] OR "vulvar tumour*"[Title/Abstract] OR "vulva tumour*"[Title/Abstract] OR "vulvar tumor*"[Title/Abstract] OR "vulva tumor*"[Title/Abstract] OR "vulvar cancer*"[Title/Abstract] OR "cancer of the vulva"[Title/Abstract] OR "vulva cancer*"[Title/Abstract] OR "cancer of vulva"[Title/Abstract] OR "vulvar squamous cell carcinom*"[Title/Abstract] OR "verrucous carcinom*"[Title/Abstract] OR "Vulvar Neoplasms"[MeSH Terms] OR "carcinoma, verrucous"[MeSH Terms:noexp] | 11.888 |

**Table S2.** Search strategy for Cochrane Library.

| **ID** | **Search** | **Hits** |
| --- | --- | --- |
| #1 | MeSH descriptor: [Vulvar Neoplasms] explode all trees | 114 |
| #2 | MeSH descriptor: [Carcinoma, Verrucous] explode all trees | 8 |
| #3 | ((vulva OR vulvar OR verrucous) NEAR/3 (malignan* or carcinom* or neoplas* or tumour* or tumor* or cancer*)):ti,ab,kw | 331 |
| #4 | (vulvar NEAR/3 (squamous NEAR/3 cell)) NEAR/3 (malignan* or carcinom* or neoplas* or tumour* or tumor* or cancer*):ti,ab,kw | 15 |
| #5 | (((vulval OR vulvar OR vulva) NEAR/3 intraepithelial) NEAR/3 (malignan* or carcinom* or neoplas* or tumour* or tumor* or cancer*)):ti,ab,kw | 83 |
| #6 | (vSCC):ti,ab,kw | 12 |
| #7 | {OR #1-#6} | 354 |
| #8 | MeSH descriptor: [Antibodies, Monoclonal, Humanized] this term only | 4839 |
| #9 | MeSH descriptor: [Immune Checkpoint Inhibitors] explode all trees | 95 |
| #10 | ("Anti-PD-L1" OR "Anti-PD-1" OR Lambrolizumab OR MK-3475 OR Keytruda OR SCH-900475 OR Pembrolizumab):ti,ab,kw | 3846 |
| #11 | ("humanized antibodies"):ti,ab,kw | 8 |
| #12 | (("programmed cell death protein“ OR "pd-1“ OR "cytotoxic t lymphocyte associated protein 4" OR "ctla 4" OR "programmed death ligand 1" OR "pd l1" OR "immune checkpoint") NEAR/3 inhibit*):ti,ab,kw | 2258 |
| #13 | ("pd 1 pd l1" OR "immune checkpoint" NEAR/3 block*):ti,ab,kw | 621 |
| #14 | {OR #8-#13} | 9977 |
| #15 | #7 AND #14 | 4 |

**Table S3.** Search strategy for Web of Science.

| **#** | **Search Query** | **Database** | **Results** |
| --- | --- | --- | --- |
| 1 | TS=((vulva OR vulvar OR verrucous) NEAR/3 (malignan* or carcinom* or neoplas* or tumour* or tumor* or cancer*)) | Web of Science Core Collection | 8175 |
| 2 | TS=((vulvar NEAR/3 (squamous NEAR/3 cell)) NEAR/3 (malignan* or carcinom* or neoplas* or tumour* or tumor* or cancer*)) | Web of Science Core Collection | 880 |
| 3 | TS=(((vulval OR vulvar OR vulva) NEAR/3 intraepithelial) NEAR/3 (malignan* or carcinom* or neoplas* or tumour* or tumor* or cancer*)) | Web of Science Core Collection | 1150 |
| 4 | TS=(vSCC) | Web of Science Core Collection | 576 |
| 5 | #4 OR #3 OR #2 OR #1 | Web of Science Core Collection | 8691 |
| 6 | TS=("Anti-PD-L1" OR "Anti-PD-1" OR Lambrolizumab OR MK-3475 OR Keytruda OR SCH-900475 OR Pembrolizumab) | Web of Science Core Collection | 24724 |
| 7 | TS=("humanized antibodies") | Web of Science Core Collection | 330 |
| 8 | TS=(("programmed cell death protein“ OR "pd-1“ OR "cytotoxic t lymphocyte associated protein 4" OR "ctla 4" OR "programmed death ligand 1" OR "pd l1" OR "immune checkpoint") NEAR/3 inhibit*) | Web of Science Core Collection | 30877 |
| 9 | TS=(("pd 1 pd l1" OR "immune checkpoint" NEAR/3 block*)) | Web of Science Core Collection | 13184 |
| 10 | #9 OR #8 OR #7 OR #6 | Web of Science Core Collection | 52816 |
| 11 | #10 AND #5 | Web of Science Core Collection | 44 |

**Table S4.** Search strategy for Clinical trials.

| Condition or disease | vulva OR vulvar OR verrucous OR vulval |
| --- | --- |
| Other Terms | Anti PD L1 OR Anti PD 1 OR Lambrolizumab OR MK-3475 OR Keytruda OR SCH-900475 OR Pembrolizumab |

**Table S5.** Search strategy for Google scholar.

|  | Vulva \| vulvar \| verrucous \| vulval malignancy \| carcinoma \| neoplasia \| neoplasm \| tumour \| tumor \| cancer Pembrolizumab \| Lambrolizumab \| MK-3475 \| Keytruda \| SCH-900475 |
| --- | --- |

# Quality assessment of the included studies

| MINORS criteria | KEYNOTE-028 | KEYNOTE-028, MINORS points |
| --- | --- | --- |
| 1 A clearly stated aim: The question addressed should be precise and relevant in the light of available literature | KEYNOTE-028 is a basket trial of 20 different cohorts of patients with PD-L1–positive, advanced solid tumors designed to assess the antitumor effects of pembrolizumab in tumor types beyond those in which clinical efficacy had been previously demonstrated | 2 |
| 2 Inclusion of consecutive patients: all patients potentially fit for inclusion (satisfying the criteria for inclusion) have been included in the study during the study period (no exclusion or details about the reasons for exclusion) | This study is a multicenter study. It is unclear if all consecutive eligible patients for each tumor entity in the different study centers were included, although the study plan required continuous enrolment. | 1 |
| 3 Prospective collection of data: data were collected according to a protocol established before the beginning of the stud | Data were collected according to a protocol. | 2 |
| 4 Endpoints appropriate to the aim of the study: unambiguous explanation of the criteria used to evaluate the main outcome, which should be in accordance with the question addressed by the study. Also, the endpoints should be assessed on an intention-to-treat basis. | The endpoints were:  -The primary endpoint: objective response rate (ORR) by IR, defined as confirmed complete response (CR) or partial response (PR) assessed per RECIST version 1.1.  -Secondary endpoints: safety, progression-free survival (PFS; time from enrollment to first documented disease progression per RECIST version 1.1 by IR or death as a result of any cause), and overall survival (OS). | 2 |
| 5 Unbiased assessment of the study endpoints: blind evaluation of objective endpoints and double-blind evaluation of subjective endpoints. Otherwise, the reasons for not blinding should be state | It is unclear if the assessment was done by blind evaluation. | 1 |
| 6 Follow-up period appropriate to the aim of the study: the follow-up should be sufficiently long to allow the assessment of the main endpoint and possible adverse events | The follow-up period for PFS and OS was longer than 20 months. | 2 |
| 7 Loss to follow-up less than 5%: all patients should be included in the follow-up. Otherwise, the proportion lost to follow-up should not exceed the proportion experiencing the major endpoint | All 18 patients entered the analyses for ORR. The study provides at least one follow-up for OS and PFS for all included patients. | 2 |
| 8 Prospective calculation of the study size: information on the size of detectable difference of interest with a calculation of 95% confidence interval, according to the expected incidence of the outcome event, and information about the level for statistical significance and estimates of power when comparing the outcomes | Sample size and power calculation were provided for ORR, accessible by Clinical Trials.org (PDF of the Study protocol) | 2 |
| Cumulative score |  | 14 |

| MINORS criteria | KEYNOTE-158 | KEYNOTE-158 |
| --- | --- | --- |
| 1 A clearly stated aim: The question addressed should be precise and relevant in the light of available literature | Study of efficacy and safety of pembrolizumab monotherapy for patients with rare, advanced solid tumors. | 2 |
| 2 Inclusion of consecutive patients: all patients potentially fit for inclusion (satisfying the criteria for inclusion) have been included in the study during the study period (no exclusion or details about the reasons for exclusion) | It is unclear if consecutive patients were included. | 1 |
| 3 Prospective collection of data: data were collected according to a protocol established before the beginning of the stud | The data were collected prospectively according to an established protocol. | 2 |
| 4 Endpoints appropriate to the aim of the study: unambiguous explanation of the criteria used to evaluate the main outcome, which should be in accordance with the question addressed by the study. Also, the endpoints should be assessed on an intention-to-treat basis. | Primary endpoint: ORR  Secondary endpoints: DOR, CR. PR, PFS, OS | 2 |
| 5 Unbiased assessment of the study endpoints: blind evaluation of objective endpoints and double-blind evaluation of subjective endpoints. Otherwise, the reasons for not blinding should be state | The ORR was evaluated by blinded independent radiologic review. | 2 |
| 6 Follow-up period appropriate to the aim of the study: the follow-up should be sufficiently long to allow the assessment of the main endpoint and possible adverse events | Follow-up period was appropriate (>20 months). | 2 |
| 7 Loss to follow-up less than 5%: all patients should be included in the follow-up. Otherwise, the proportion lost to follow-up should not exceed the proportion experiencing the major endpoint | All patients entered the analyses for ORR. | 2 |
| 8 Prospective calculation of the study size: information on the size of detectable difference of interest with a calculation of 95% confidence interval, according to the expected incidence of the outcome event, and information about the level for statistical significance and estimates of power when comparing the outcomes | The study size was prospectively calculated for ORR with 95% CI. | 2 |
| Cumulative score |  | 15 |

# Funnel Plots


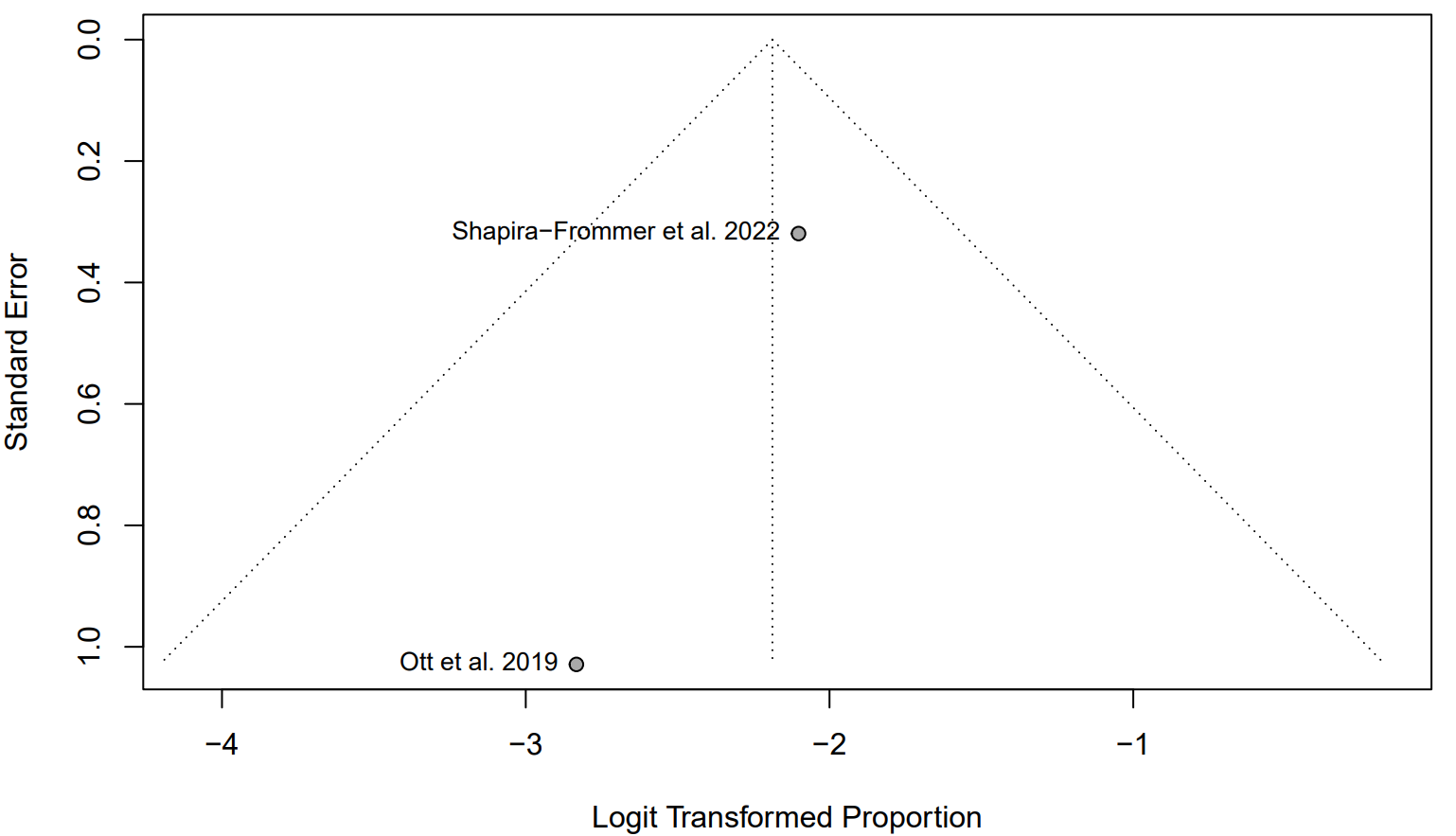


**Figure S1.** Funnel plot, ORR in total population.


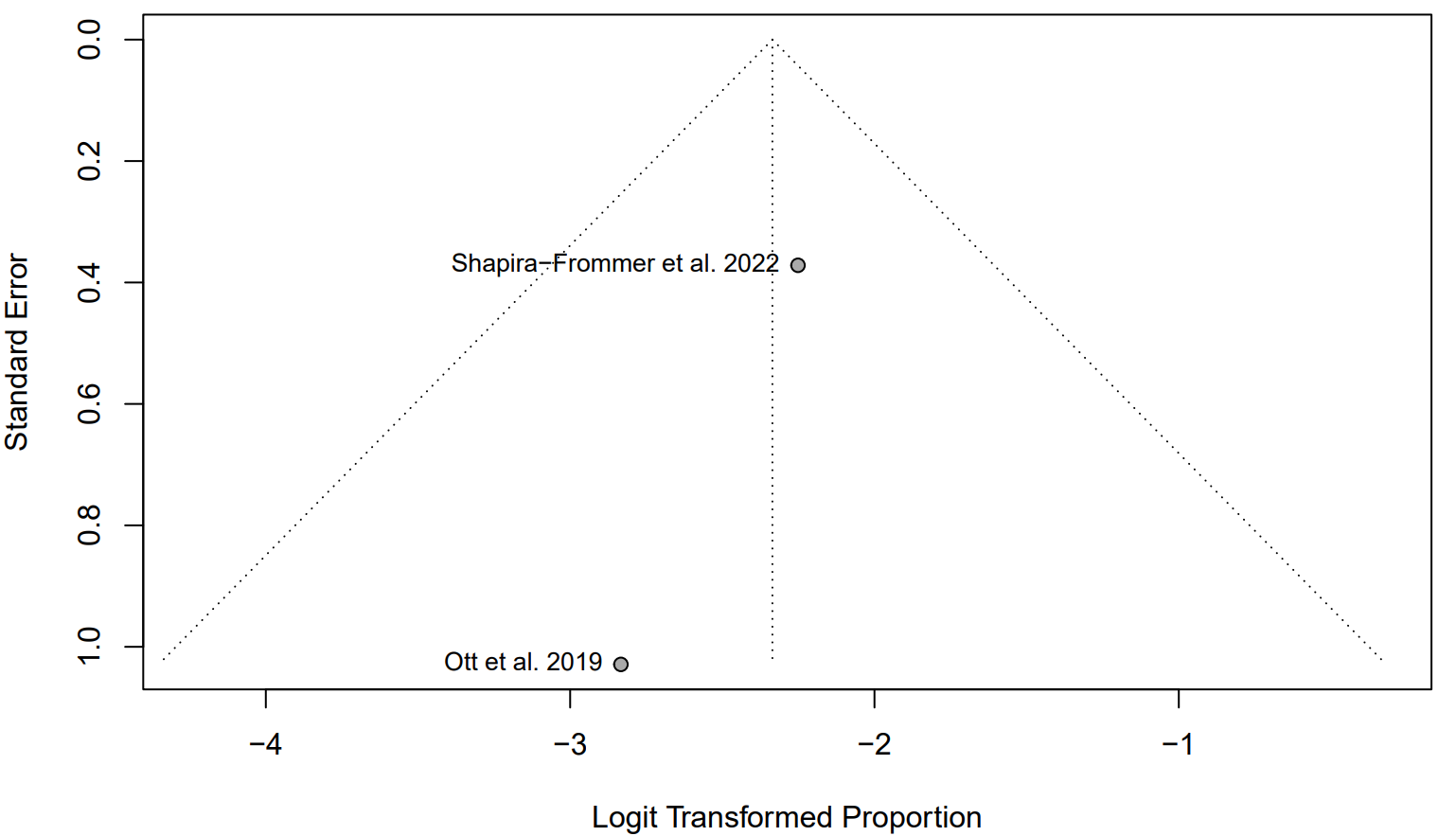


**Figure S2.** Funnel plot, ORR PD-L1 positive population.


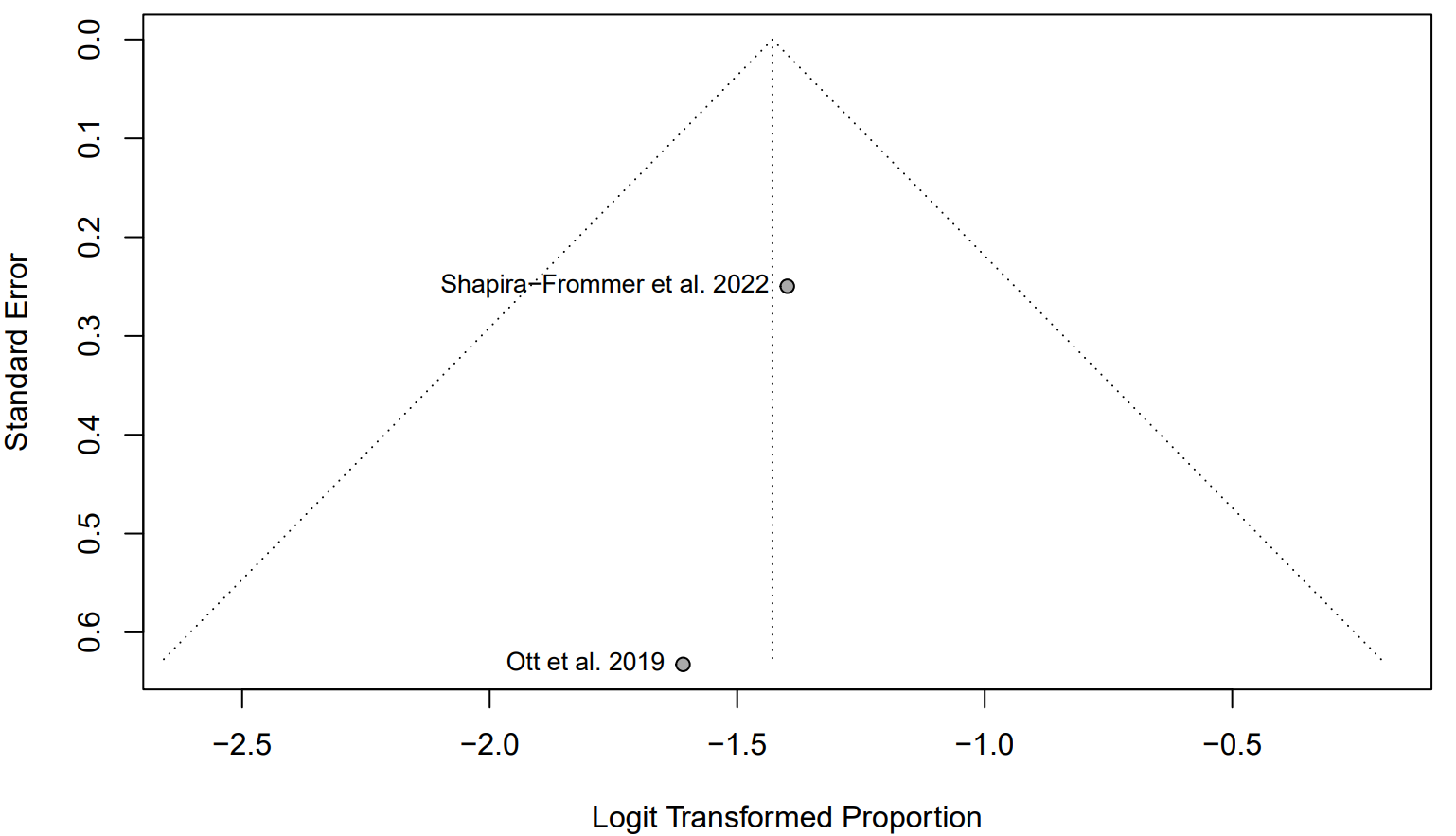


**Figure S3.** Funnel plot, PFS, ITT, 6 months.


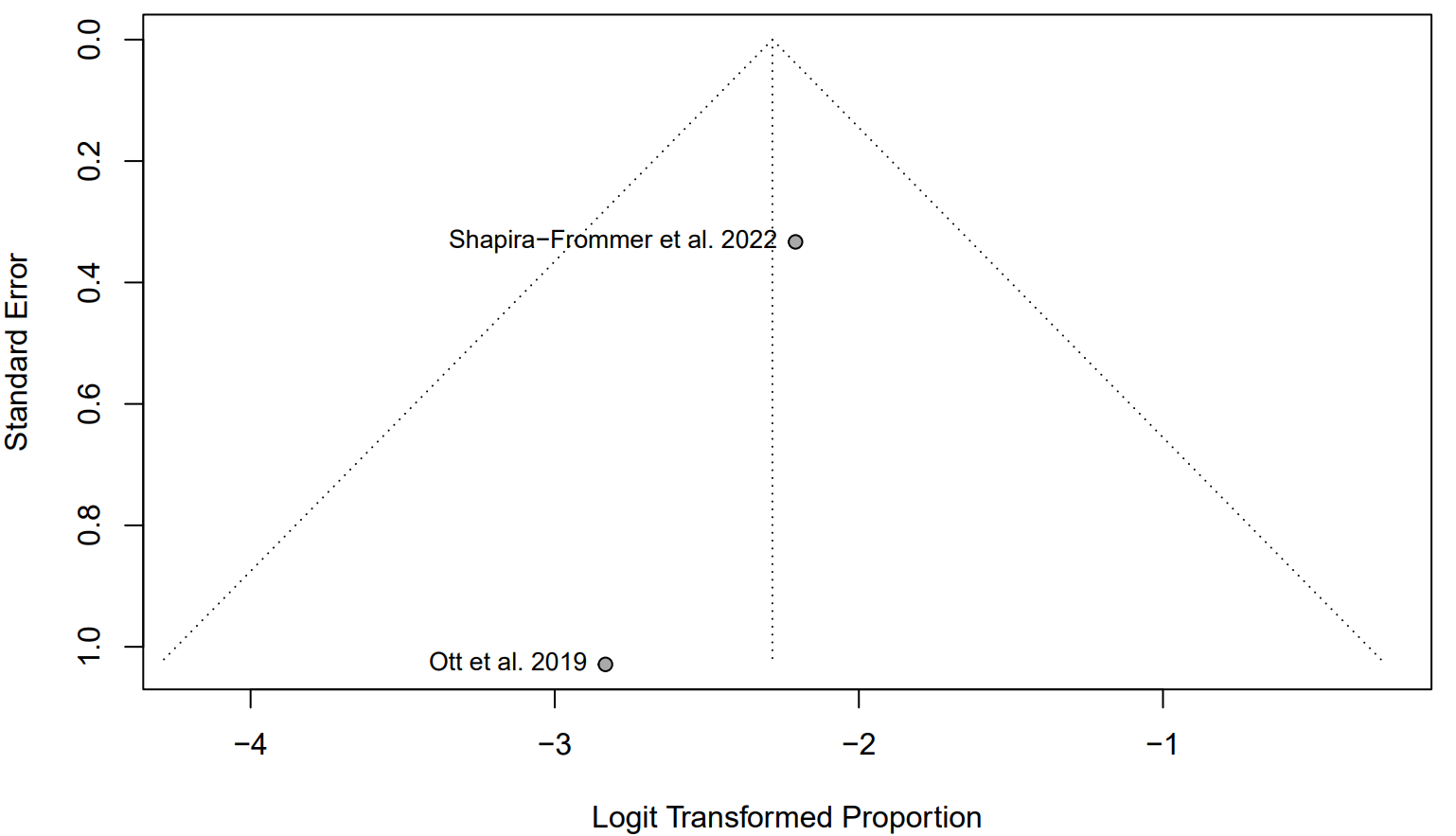


**Figure S4.** Funnel plot, PFS, ITT, 12 months.


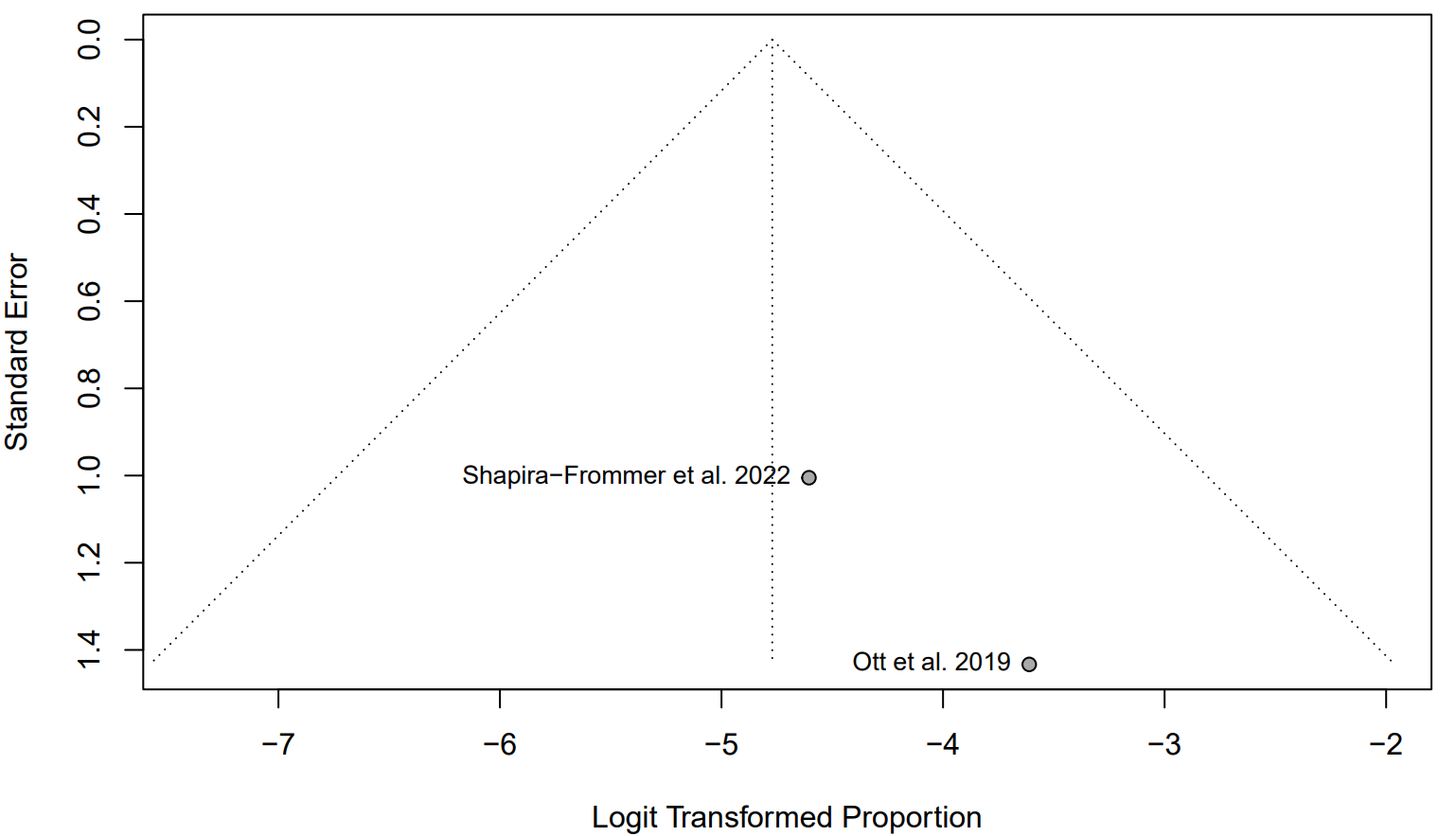


**Figure S5.** Funnel plot, PFS, ITT, 24 months.


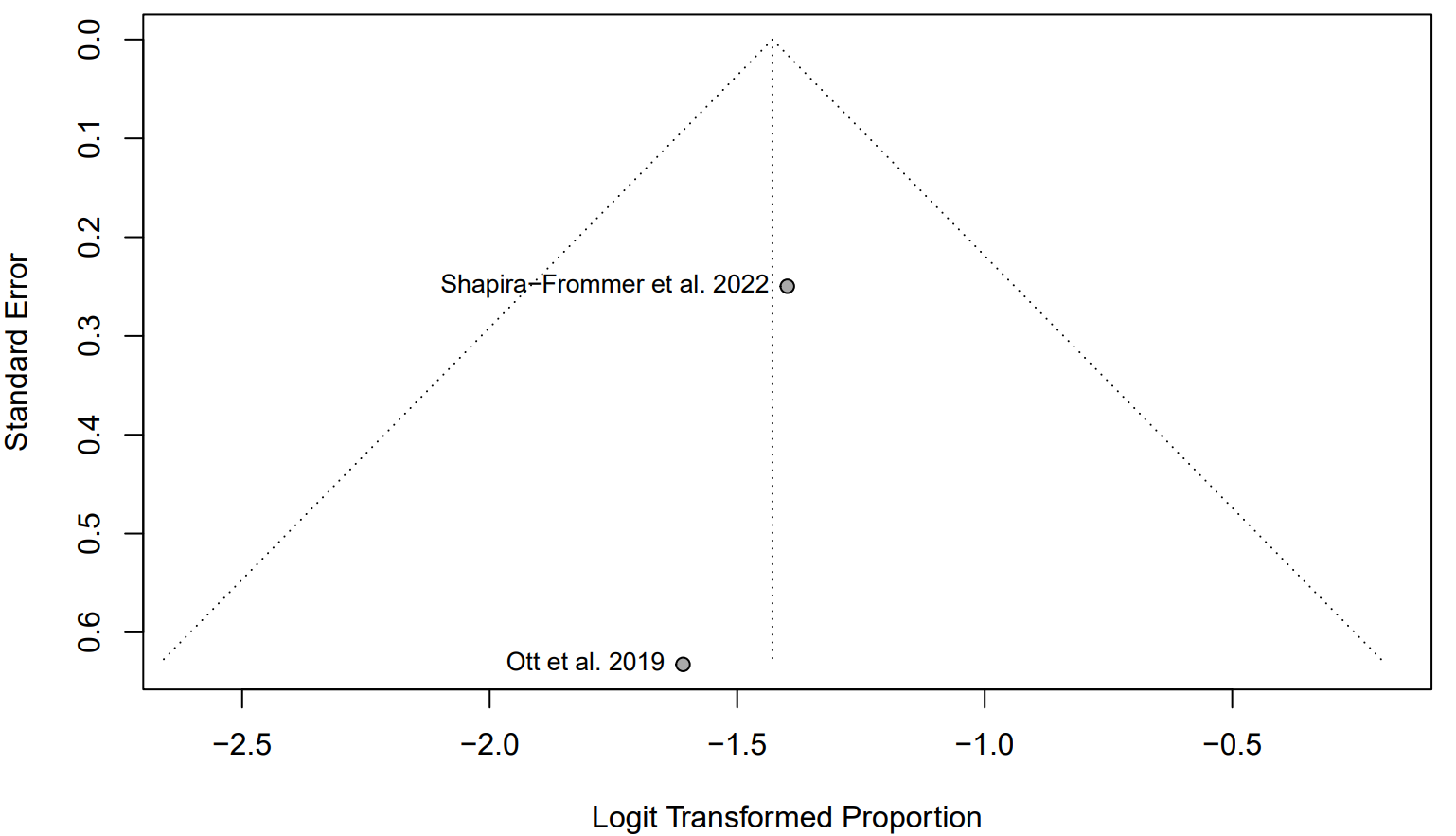


**Figure S6.** Funnel plot, PFS, PPA, 6 months.


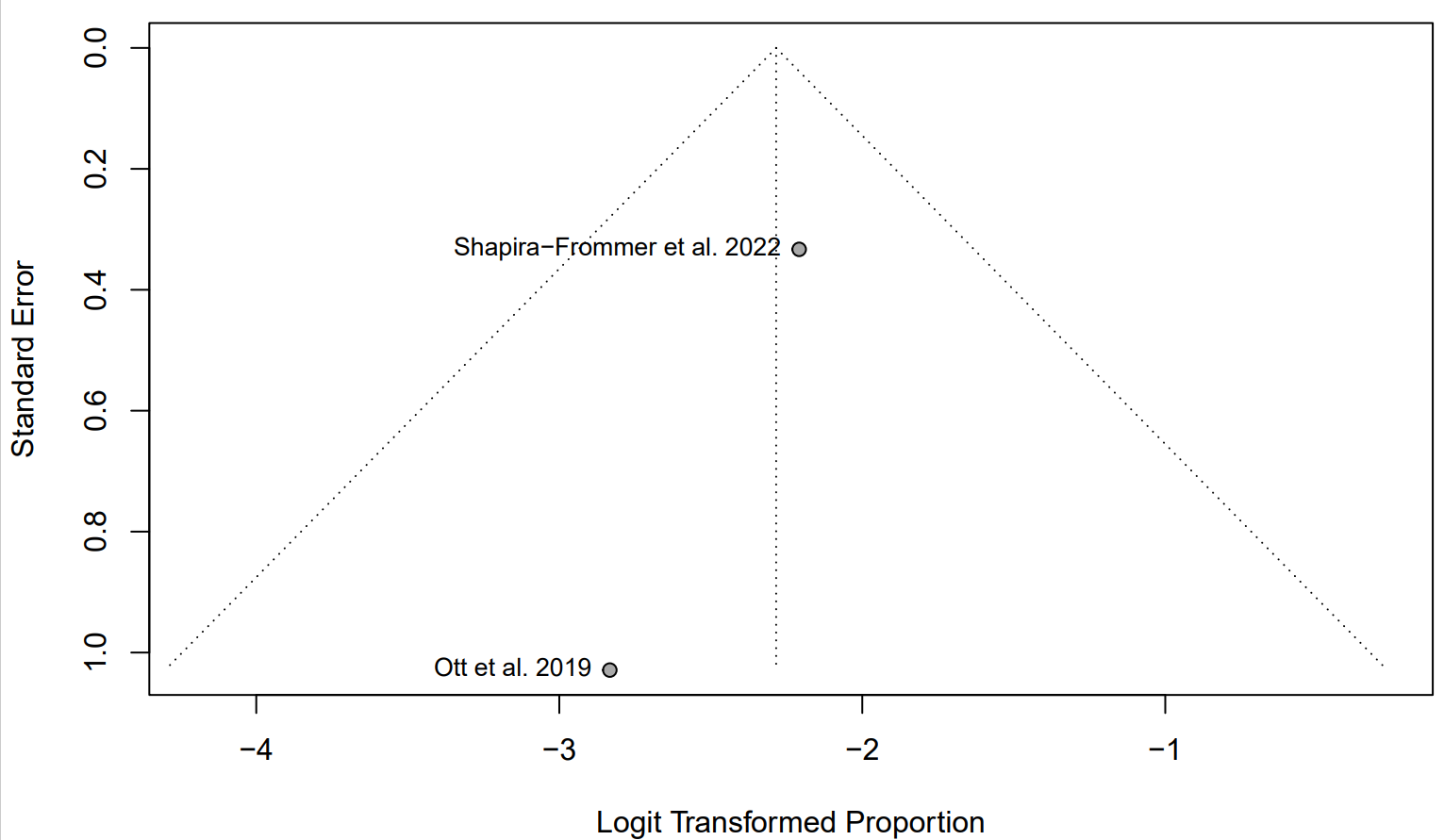


**Figure S7.** Funnel plot, PFS, PPA, 12 months.


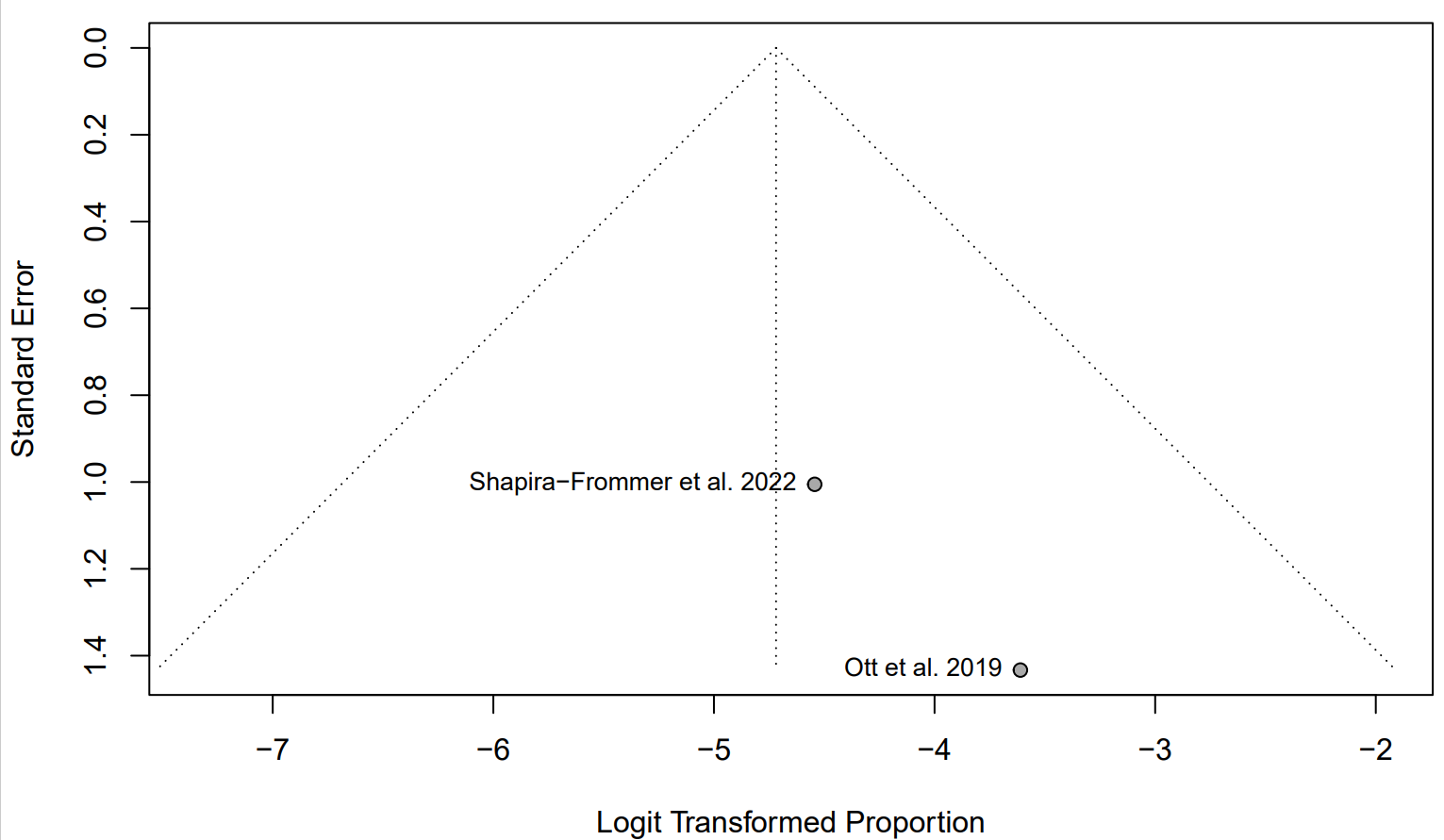


**Figure S8.** Funnel plot, PFS, PPA, 24 monhts.


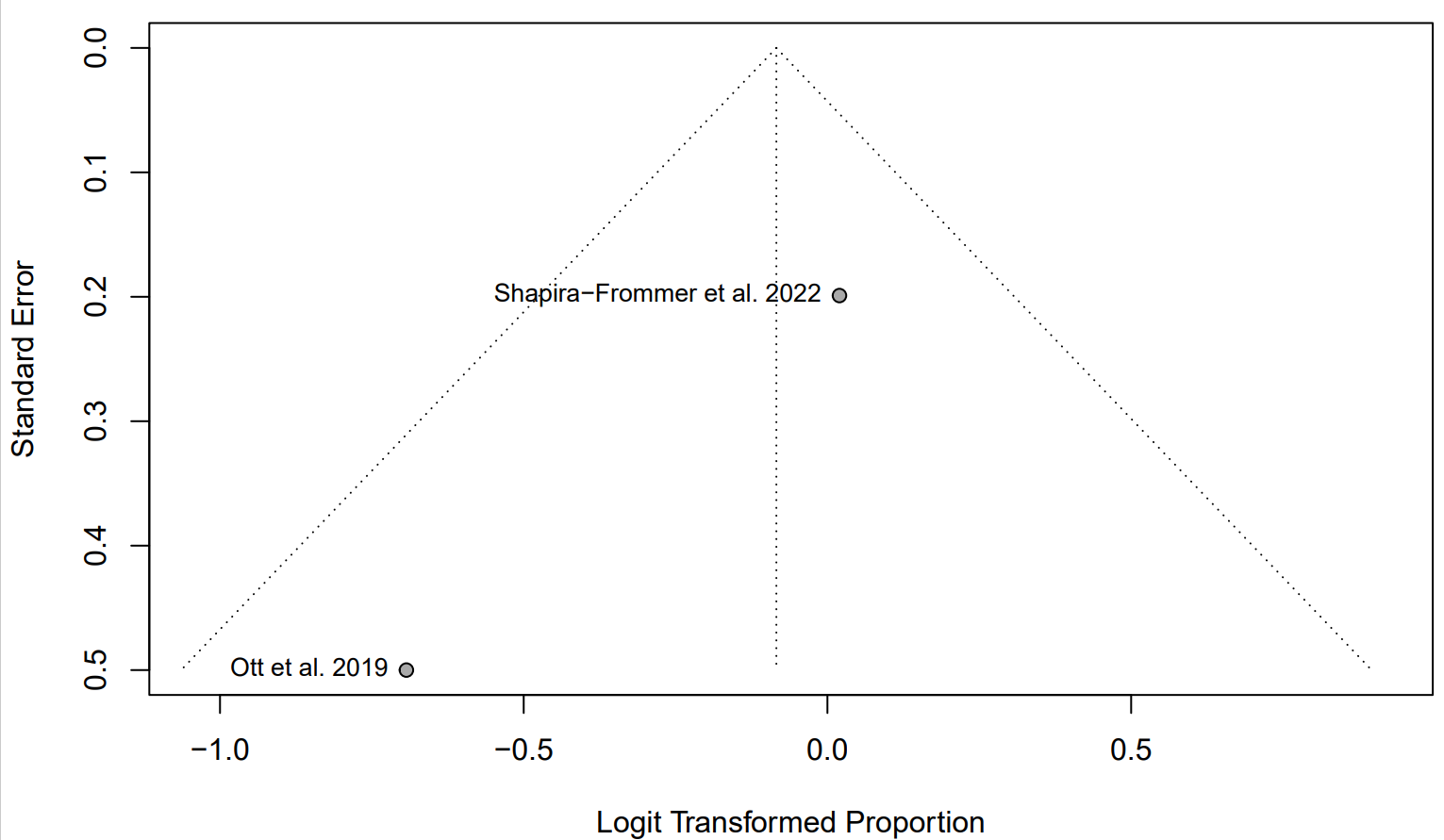


**Figure S9.** Funnel plot, OS, ITT 6 months.


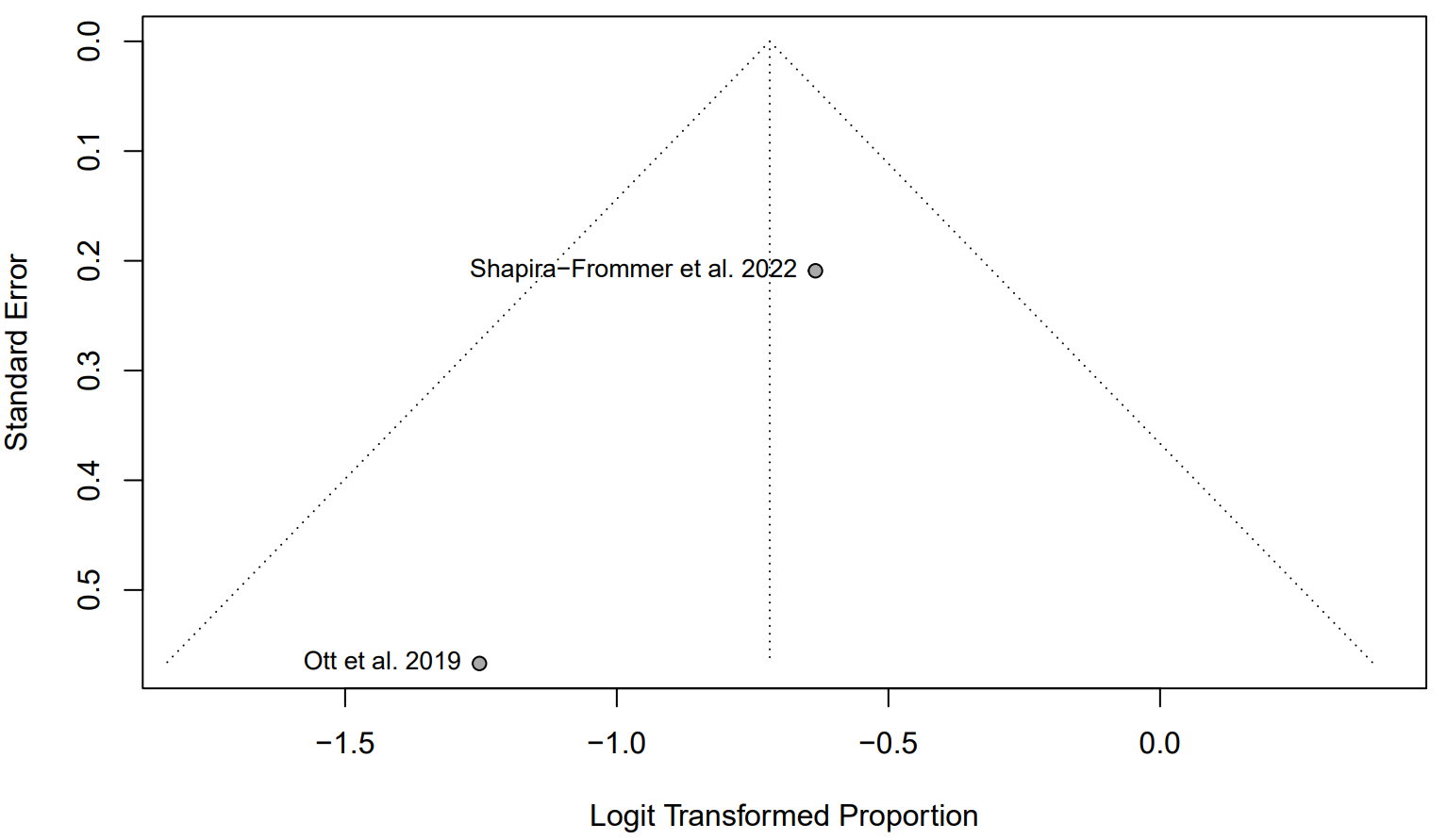


**Figure S10.** OS, ITT, 12 months.


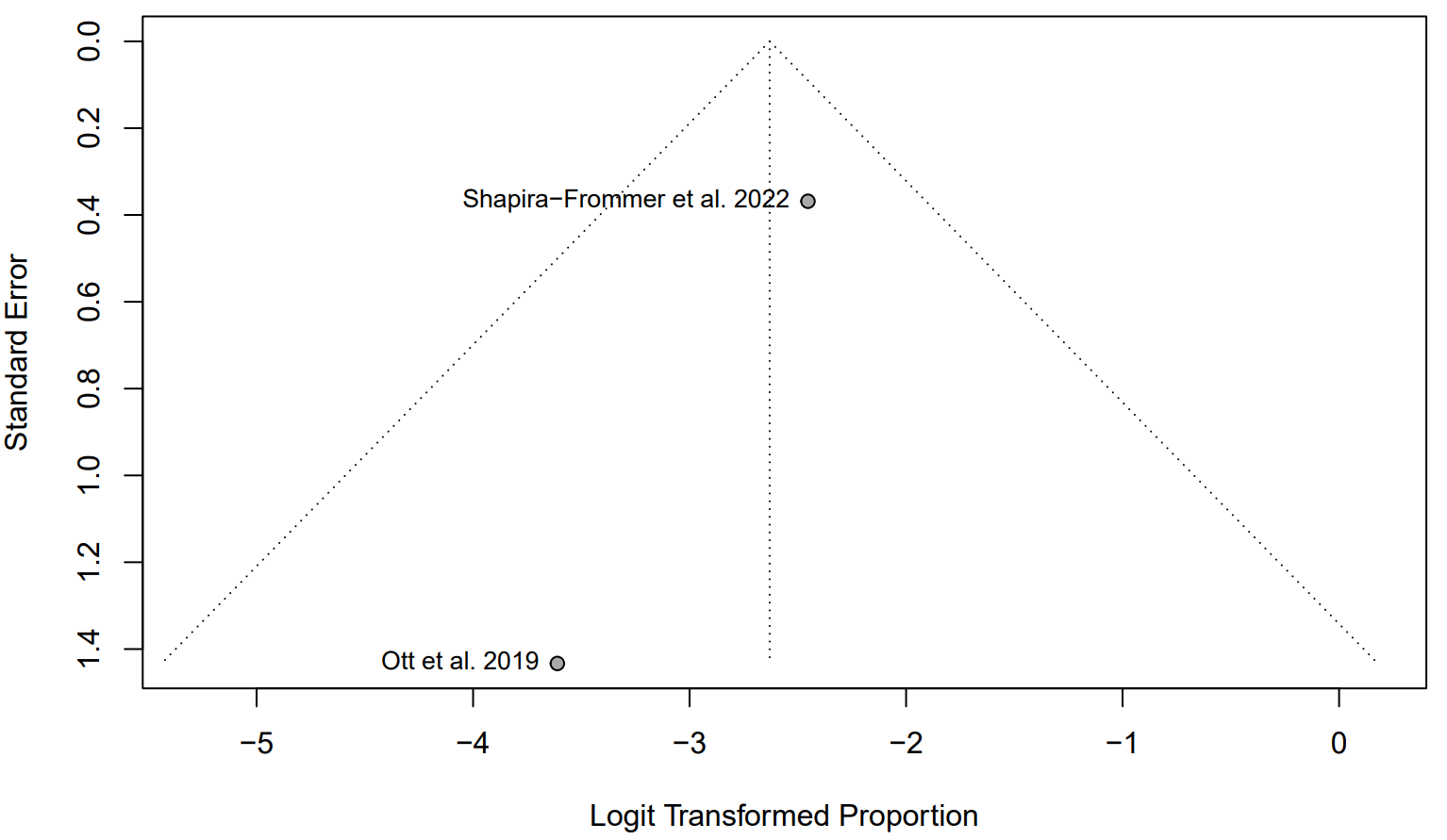


**Figure S11.** Funnel plot, OS, ITT, 24 months.


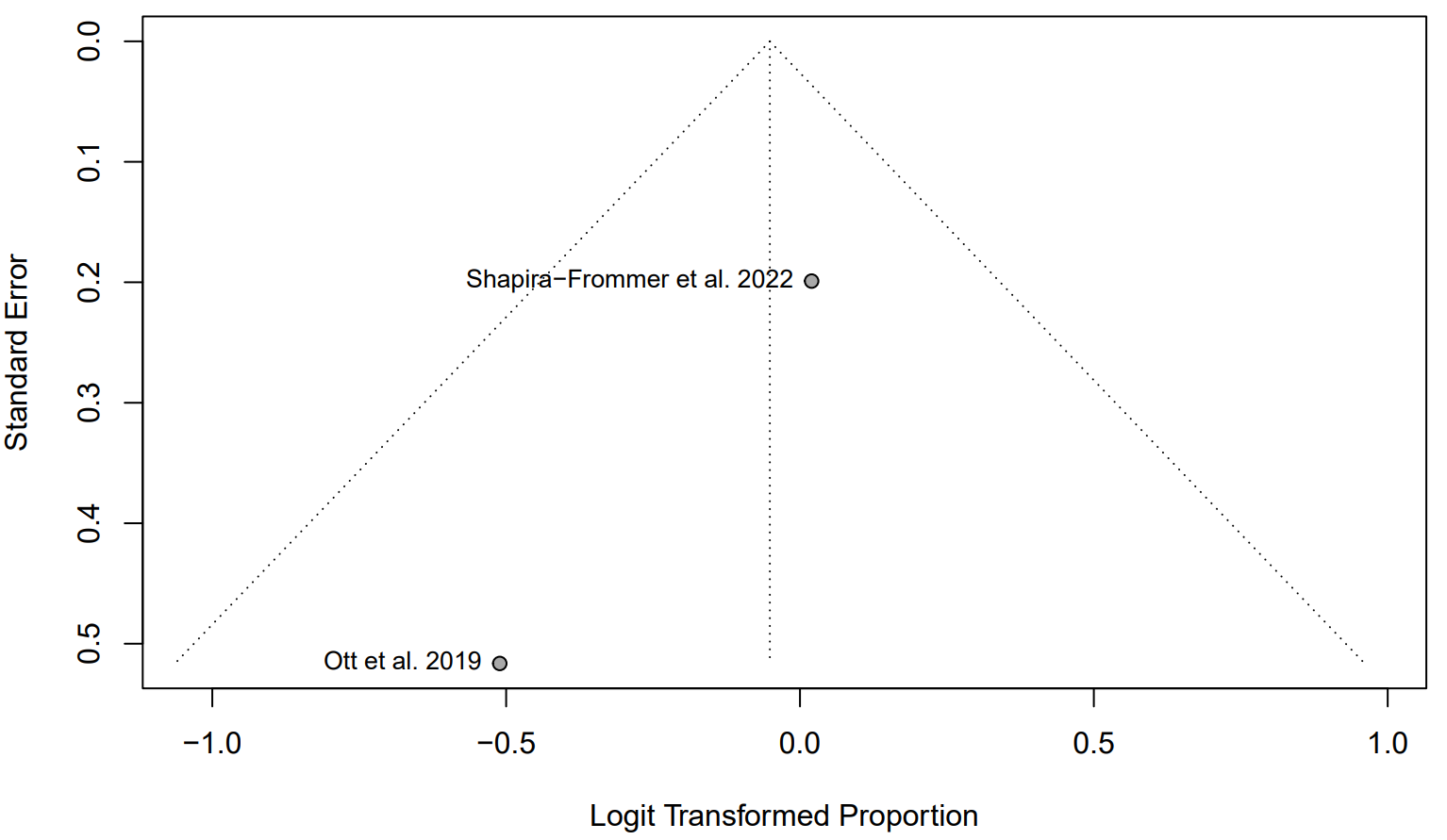


**Figure S12.** Funnel plot, OS, PPA, 6 months.


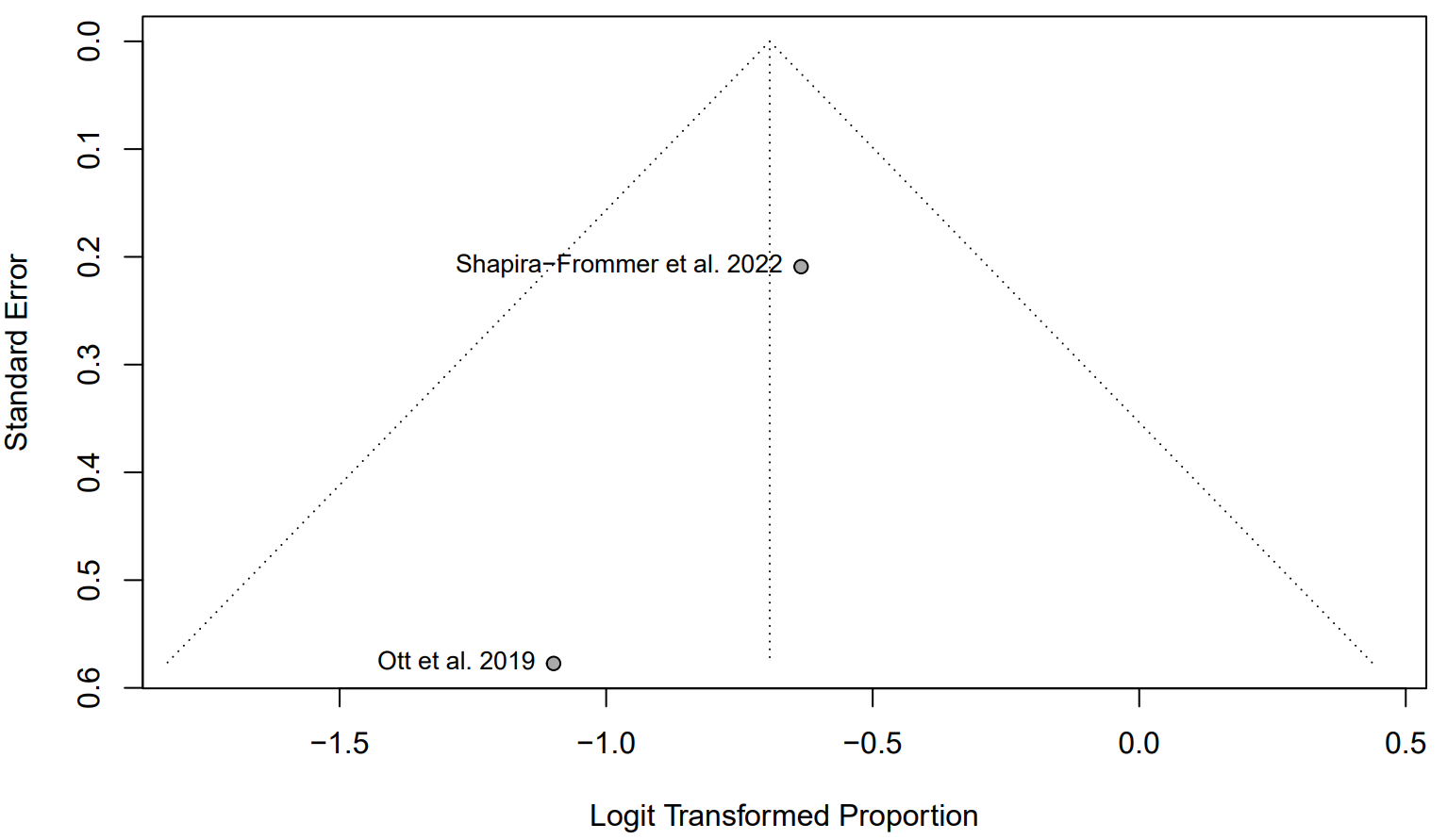


**Figure S13.** Funnel plot, OS, PPA, 12 months.


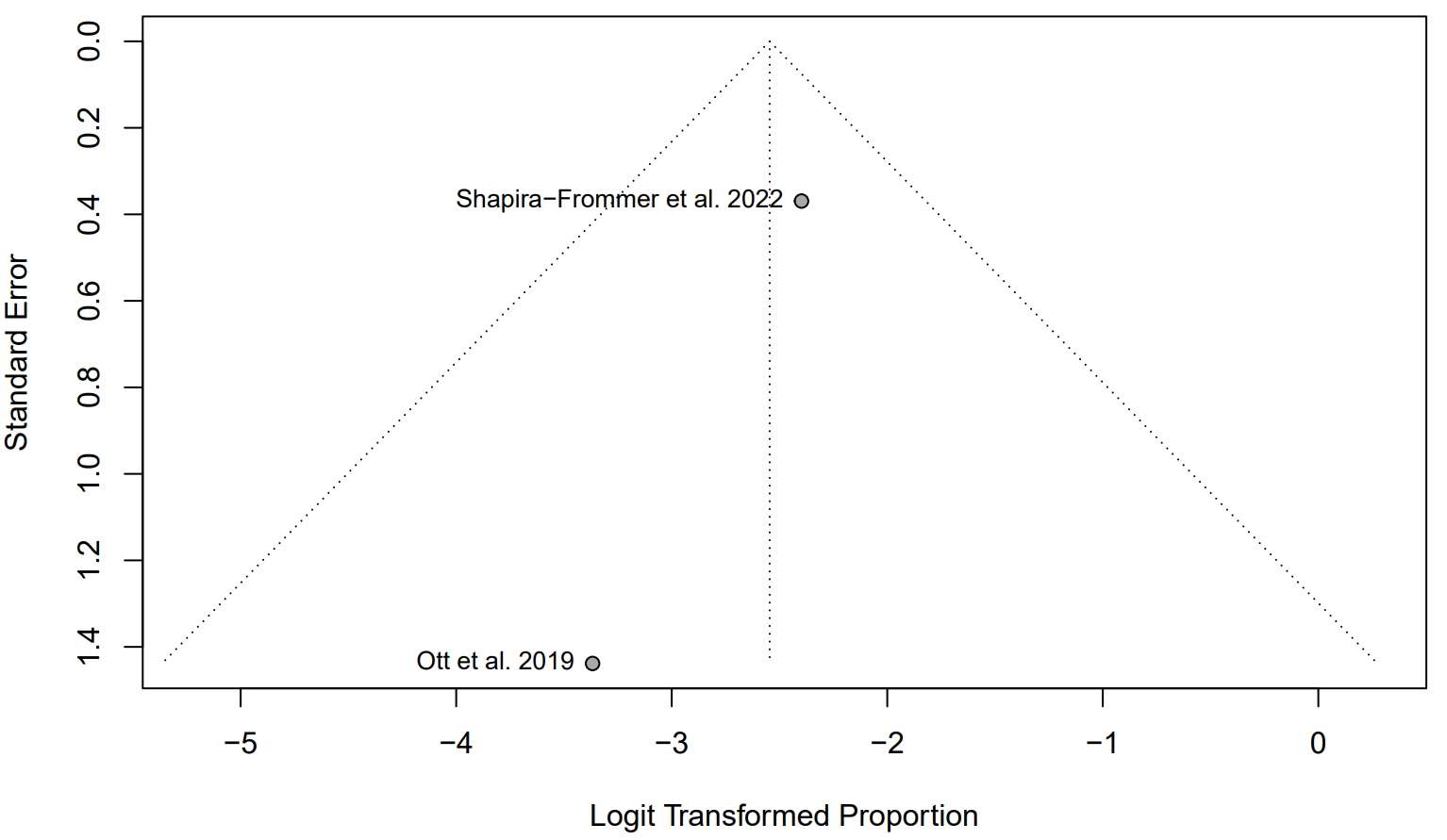


**Figure S14**. Funnel plot, OS, PPA, 24 months.


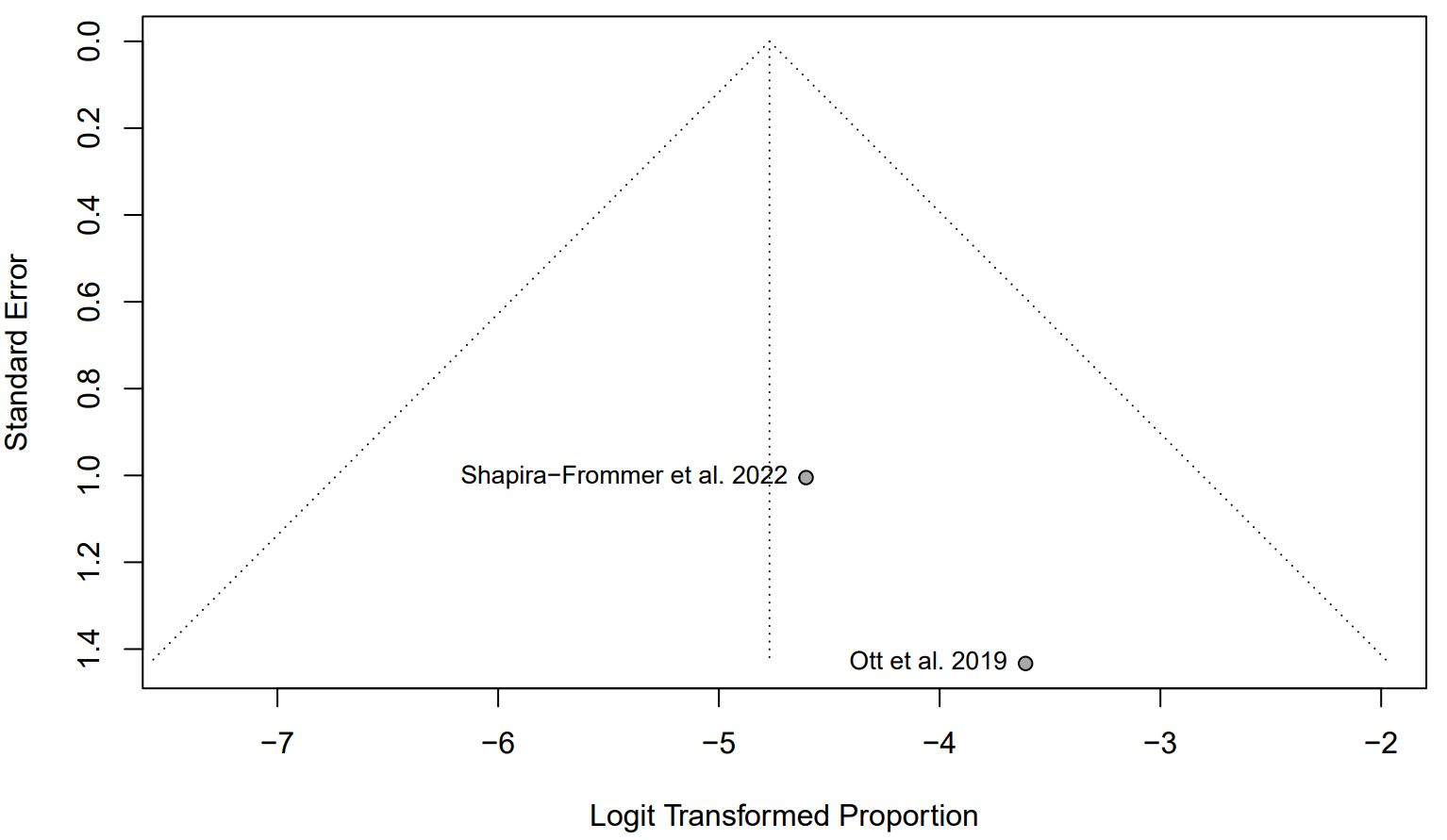


**Figure S15.** Funnel plot, Complete response, ITT.


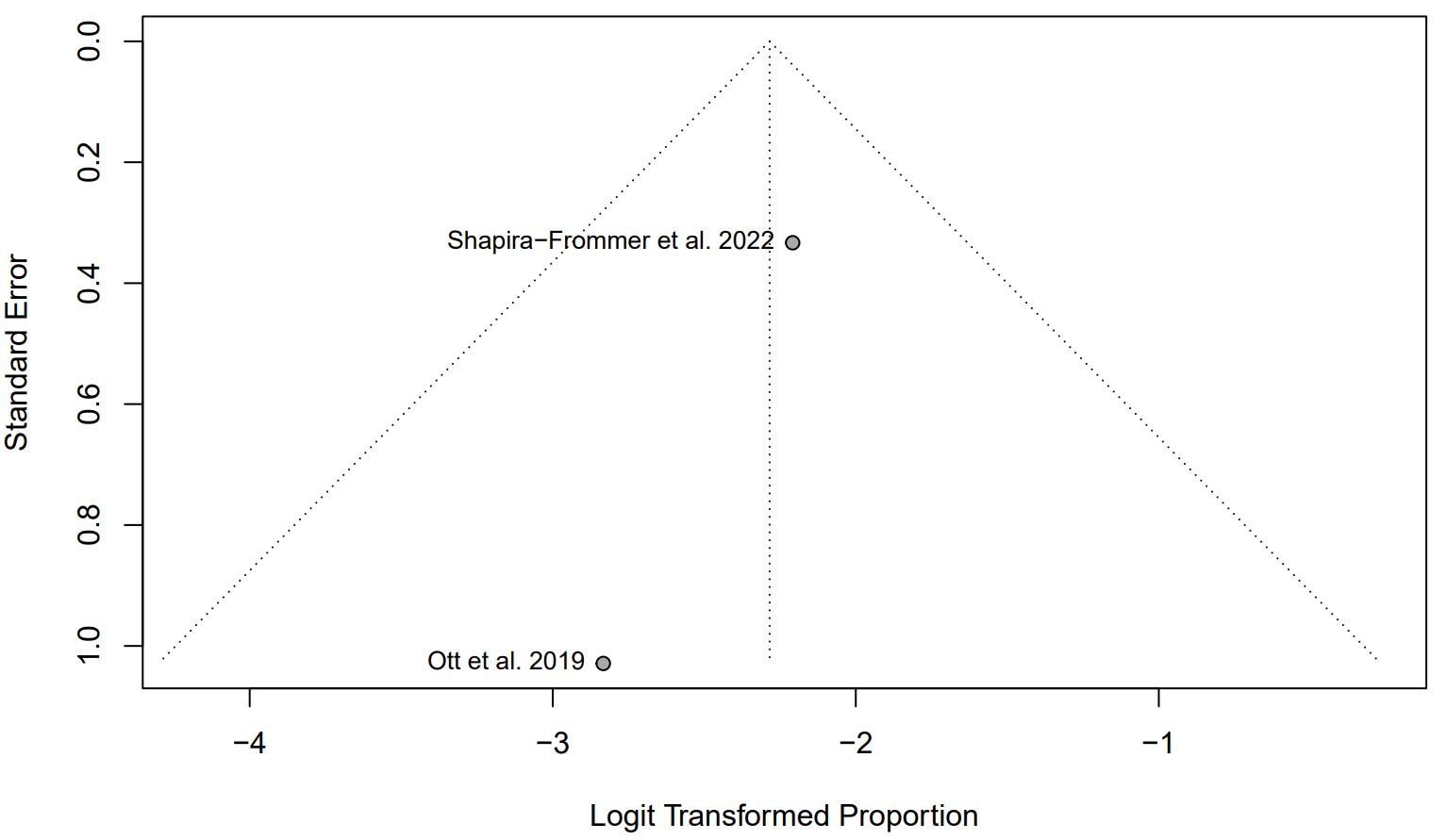


**Figure S16.** Funnel plot, Partial response, ITT.


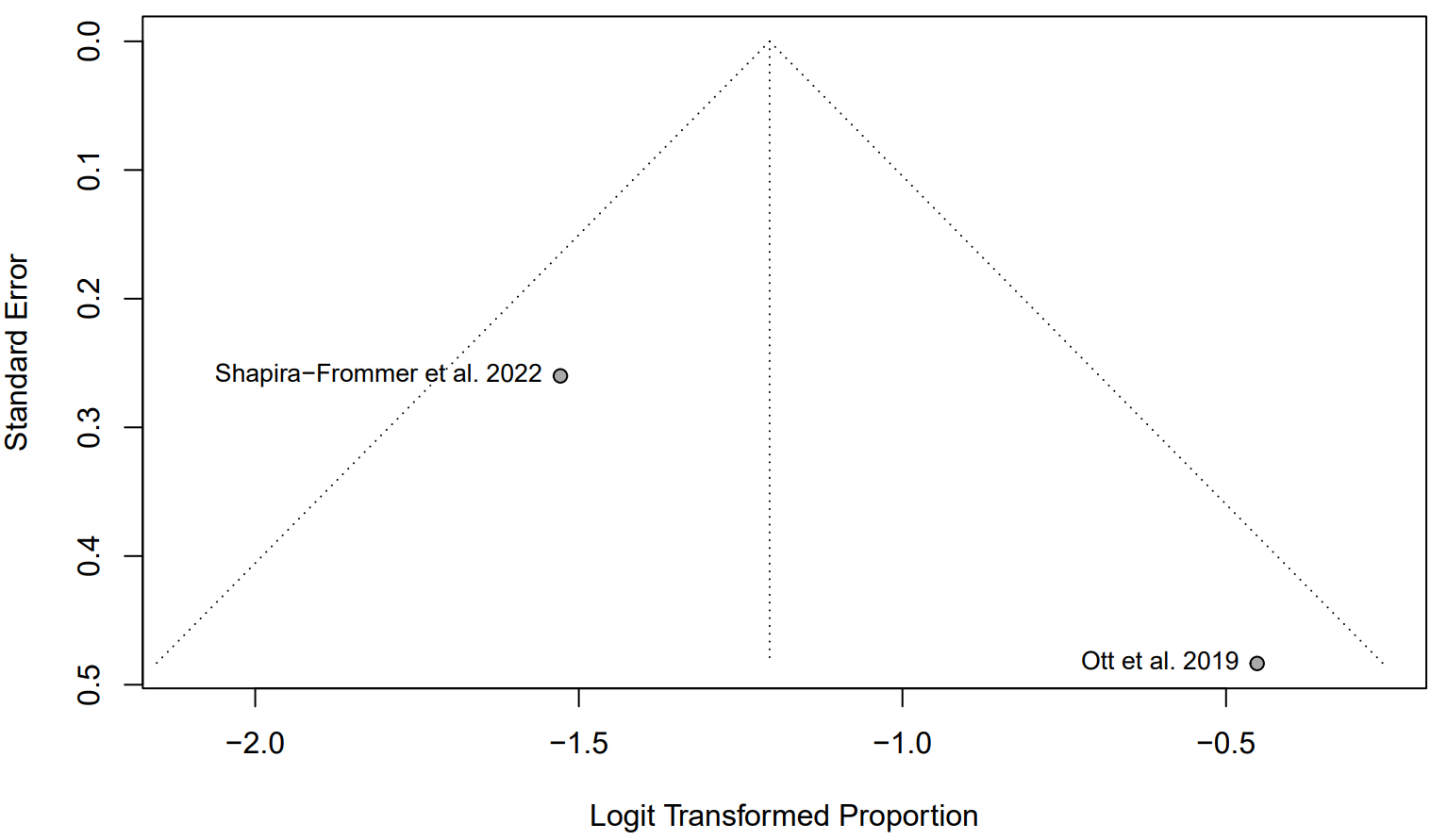


**Figure S17.** Funnel plot, Stable disease, ITT.


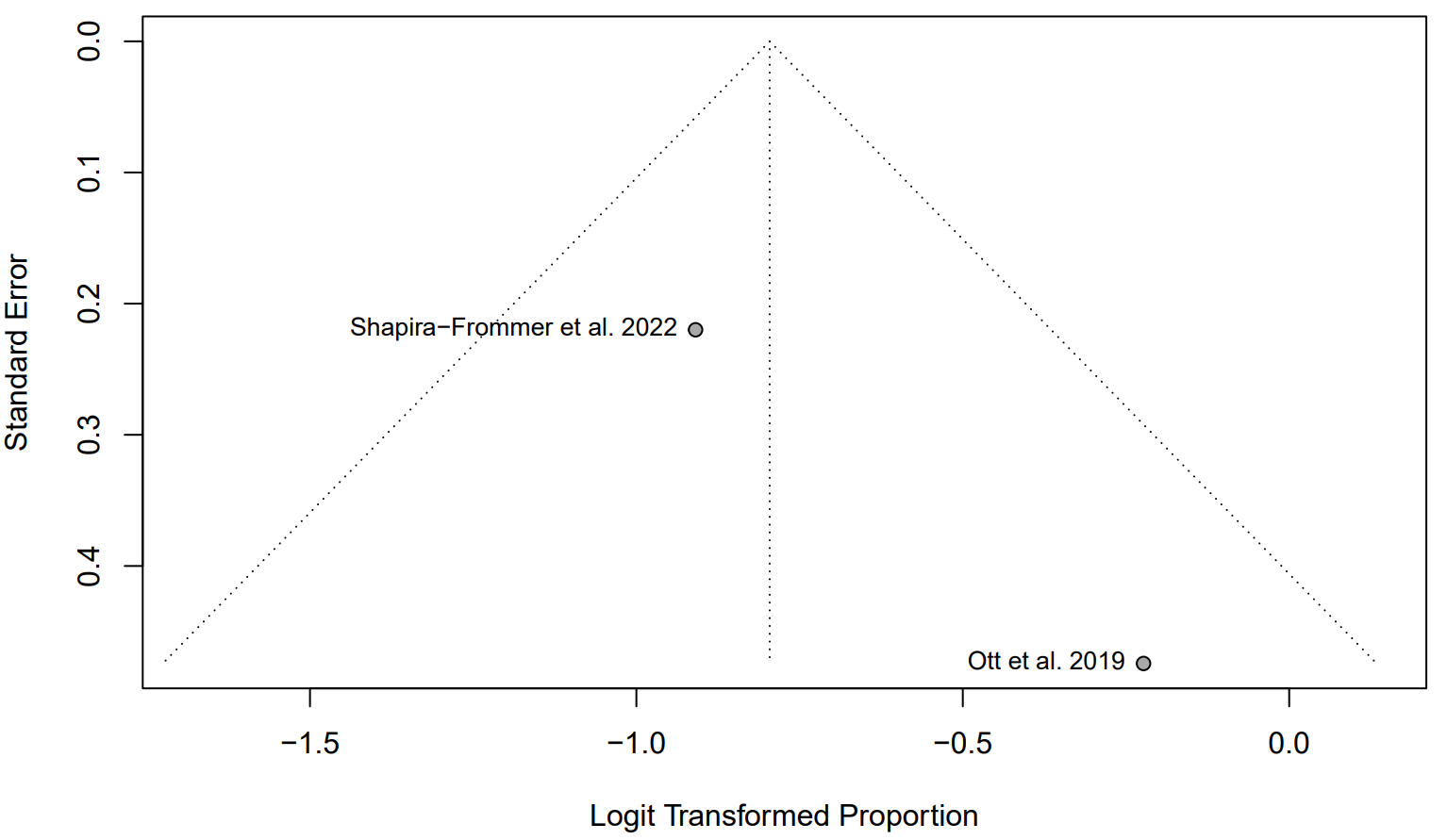


**Figure S18.** Funnel plot, Any benefit, ITT.


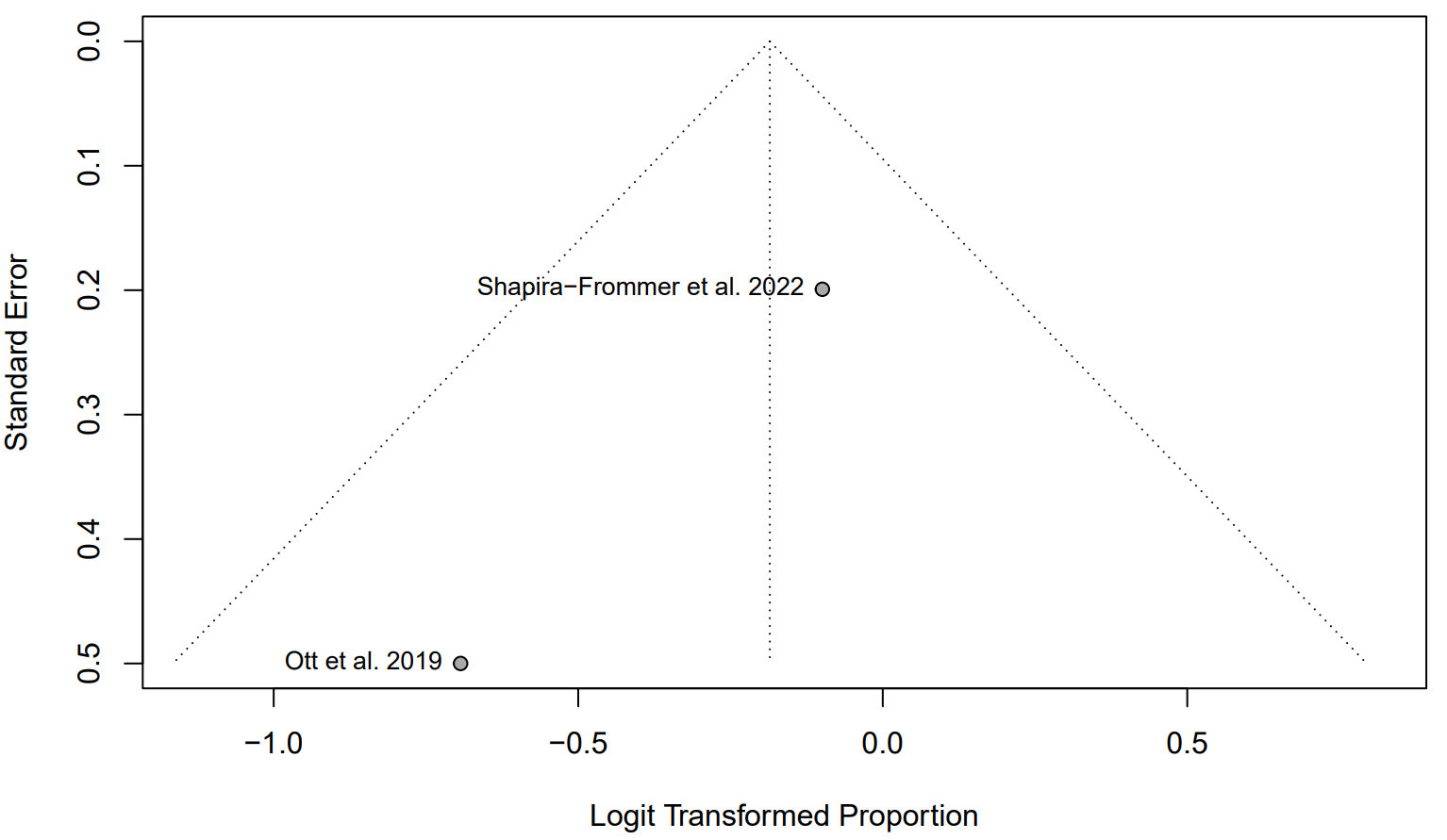


**Figure S19.** Funnel plot, Progressive disease, ITT.


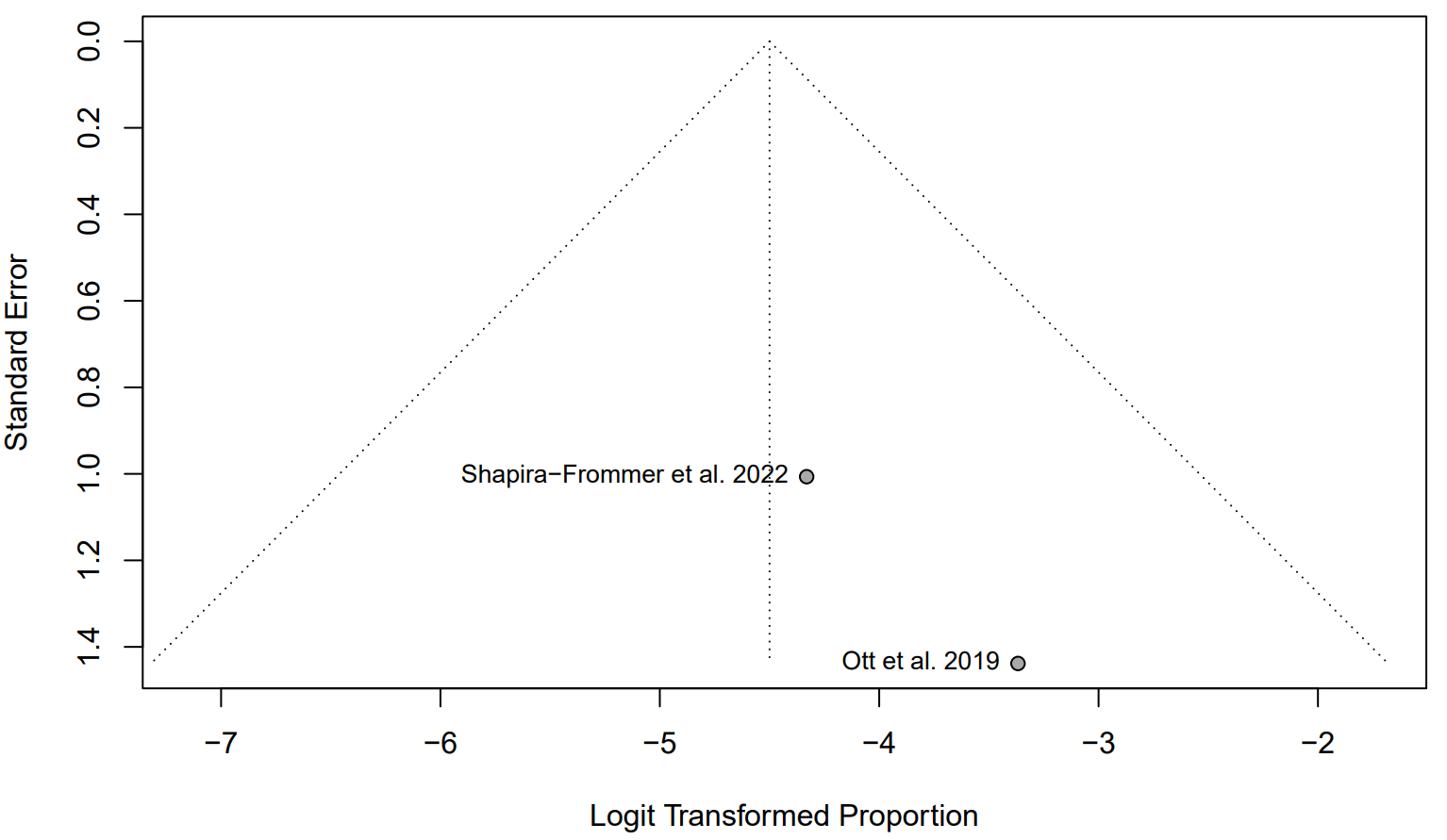


**Figure S20.** Funnel plot, Complete response, PPA.


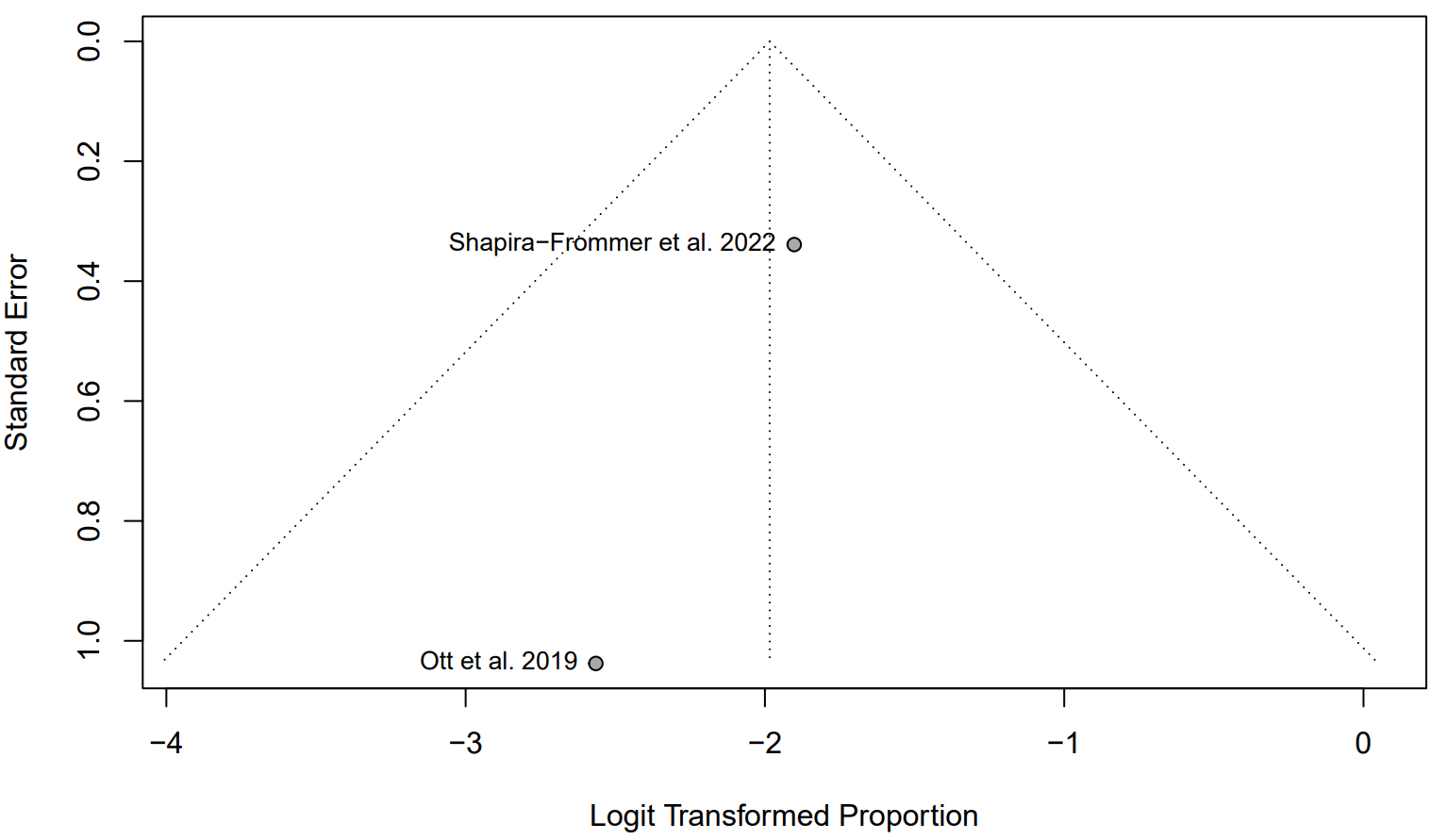


**Figure S21.** Funnel plot, Partial response, PPA.


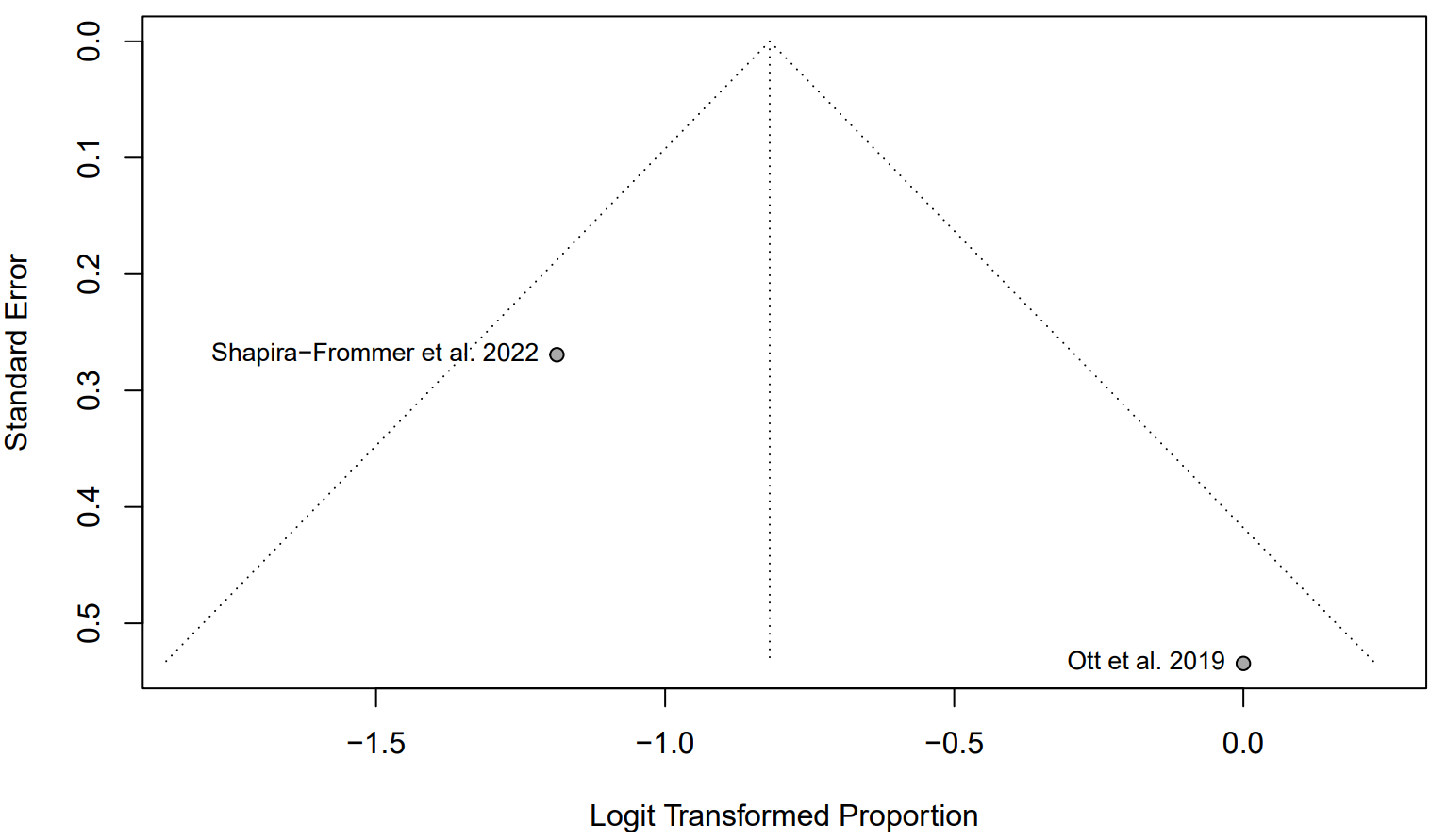


**Figure S22.** Funnel plot, Stable disease, PPA.


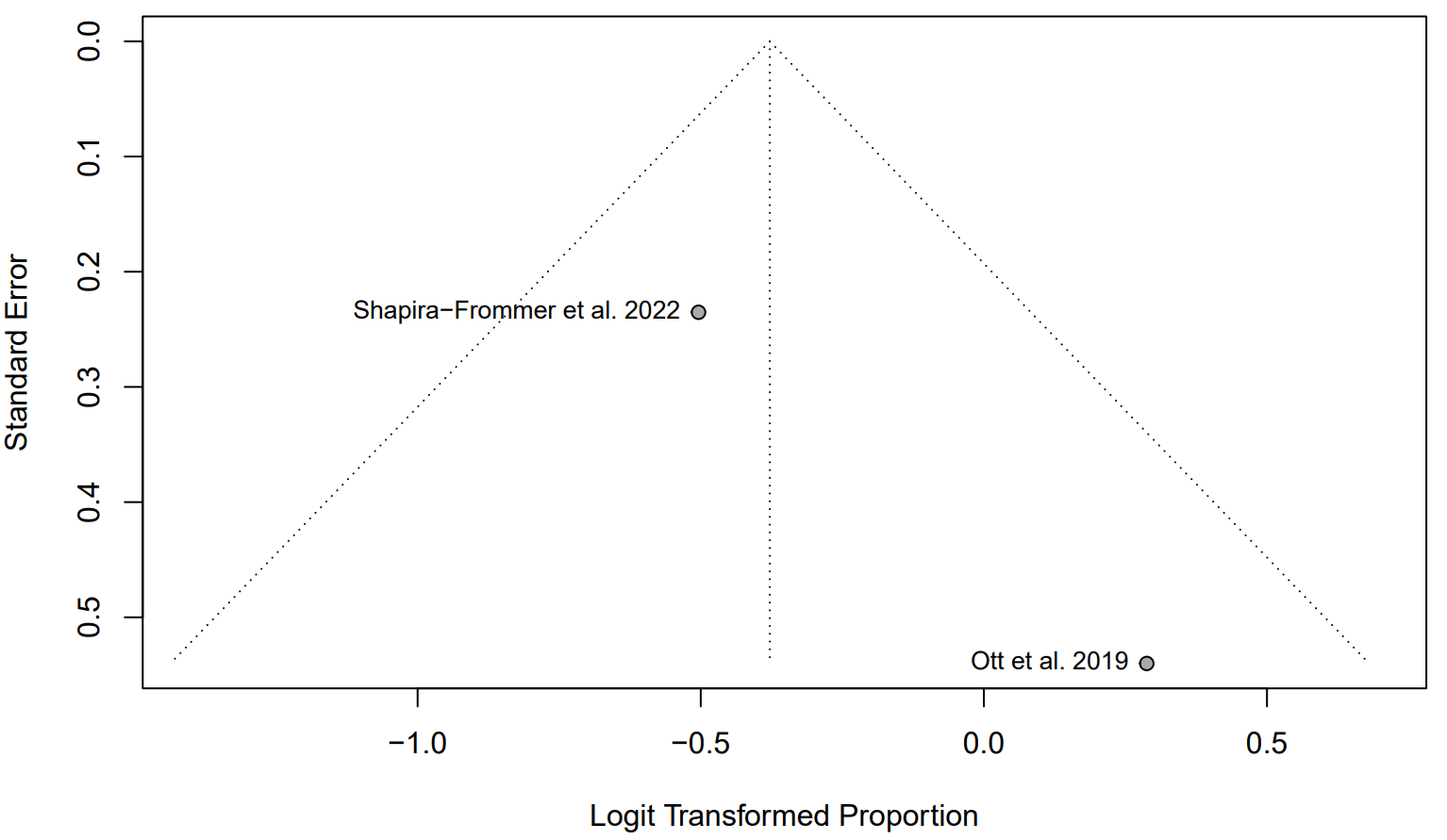


**Figure S23.** Funnel plot, Any benefit, PPA.


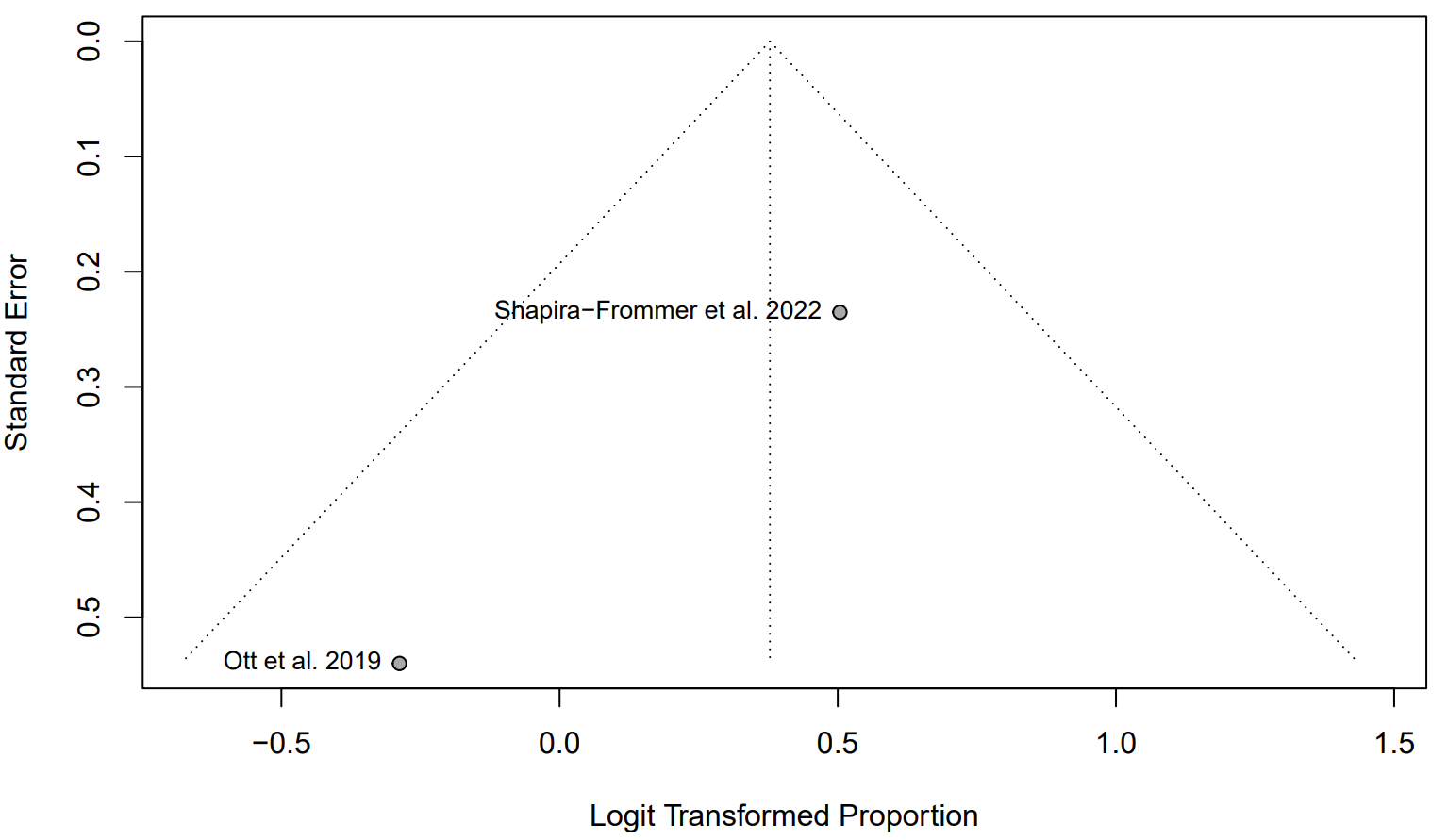


**Figure S24.** Funnel plot, Progressive disease, PPA.

# Baujat Plots


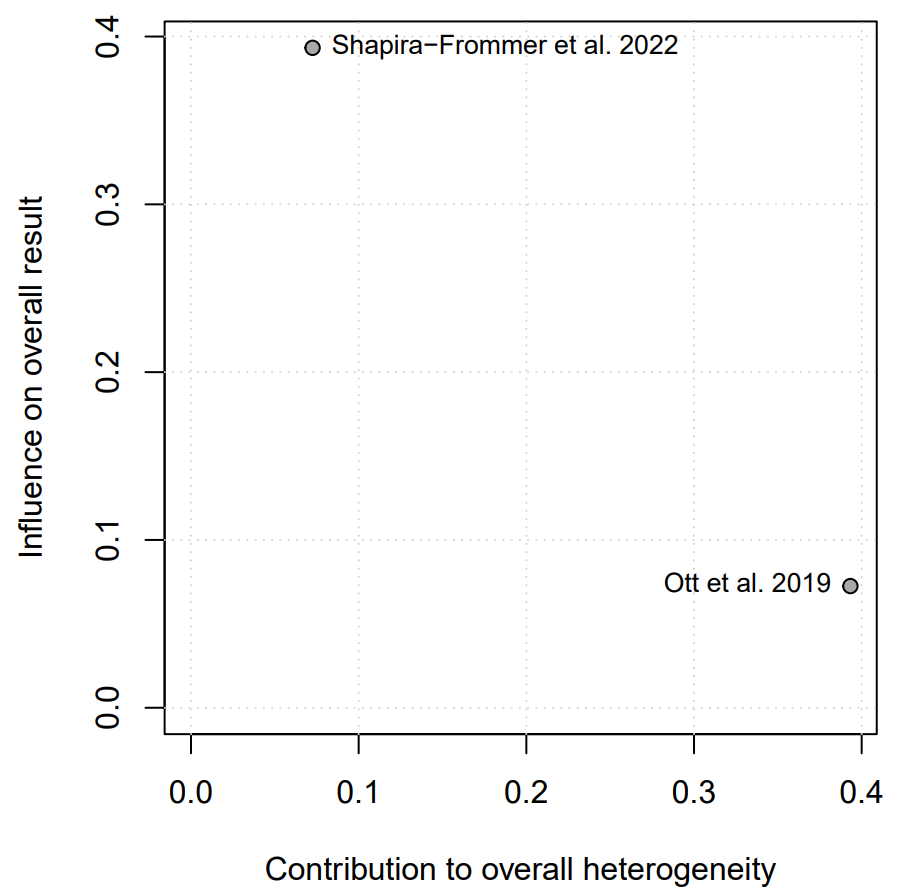


**Figure S25.** Baujat plot, ORR, total population.


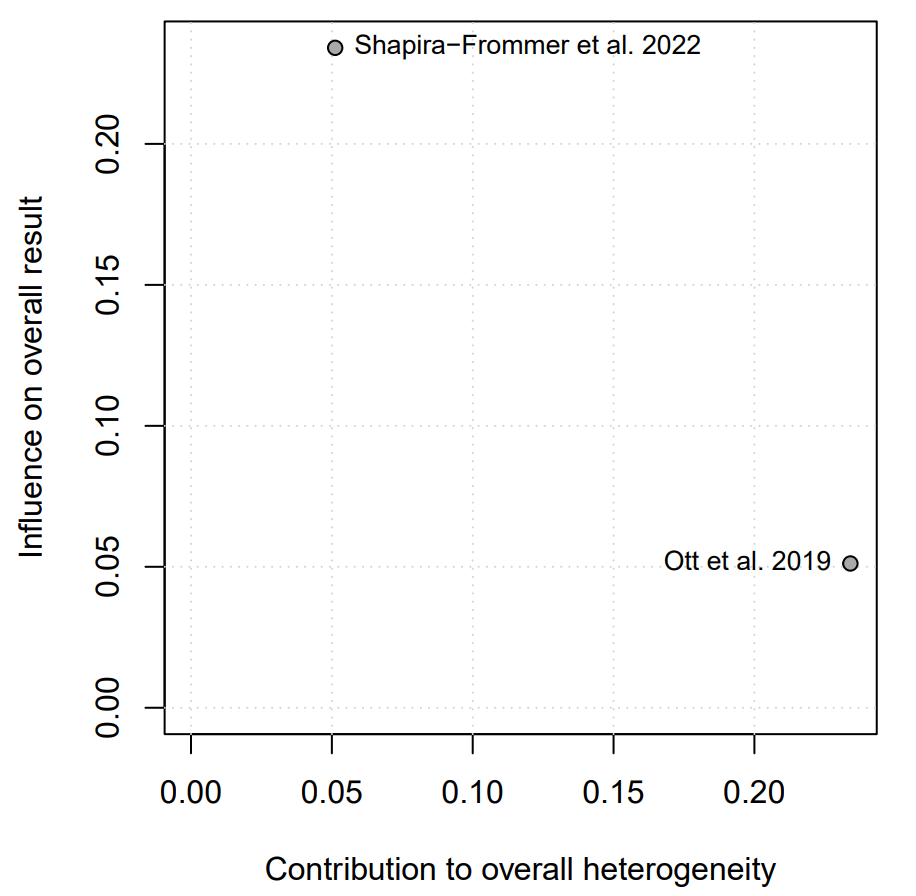


**Figure S26.** Baujat plot, ORR, PD-L1 positive population.


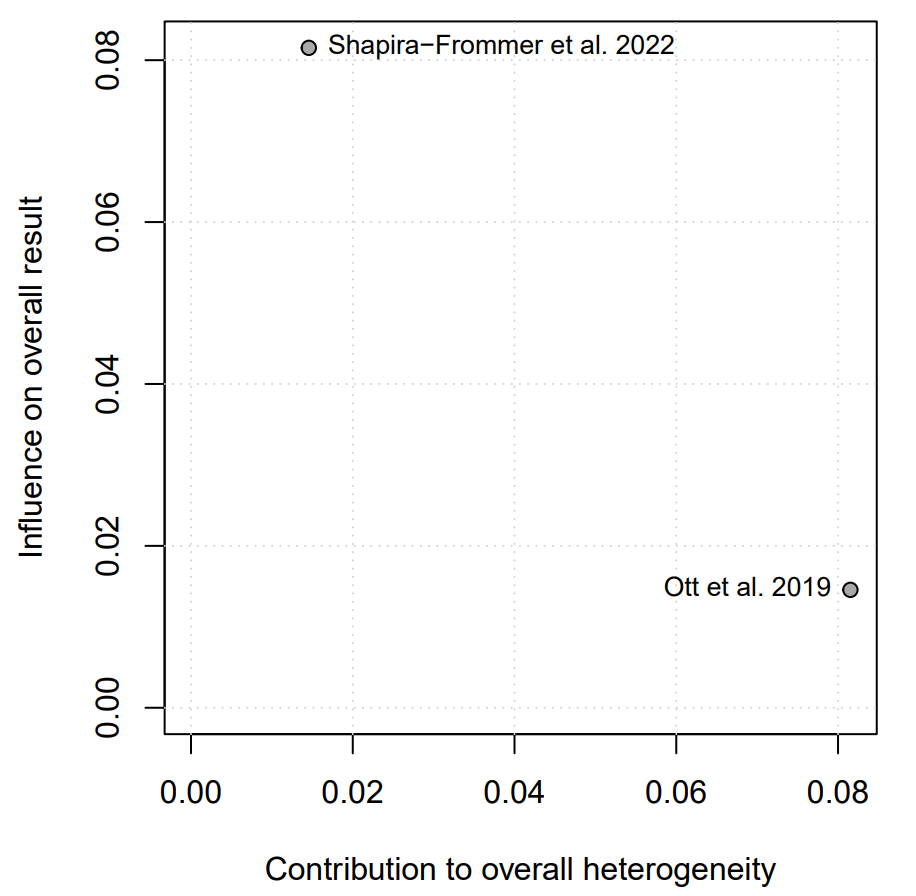


**Figure S27.** Baujat plot, PFS, ITT, 6 months.


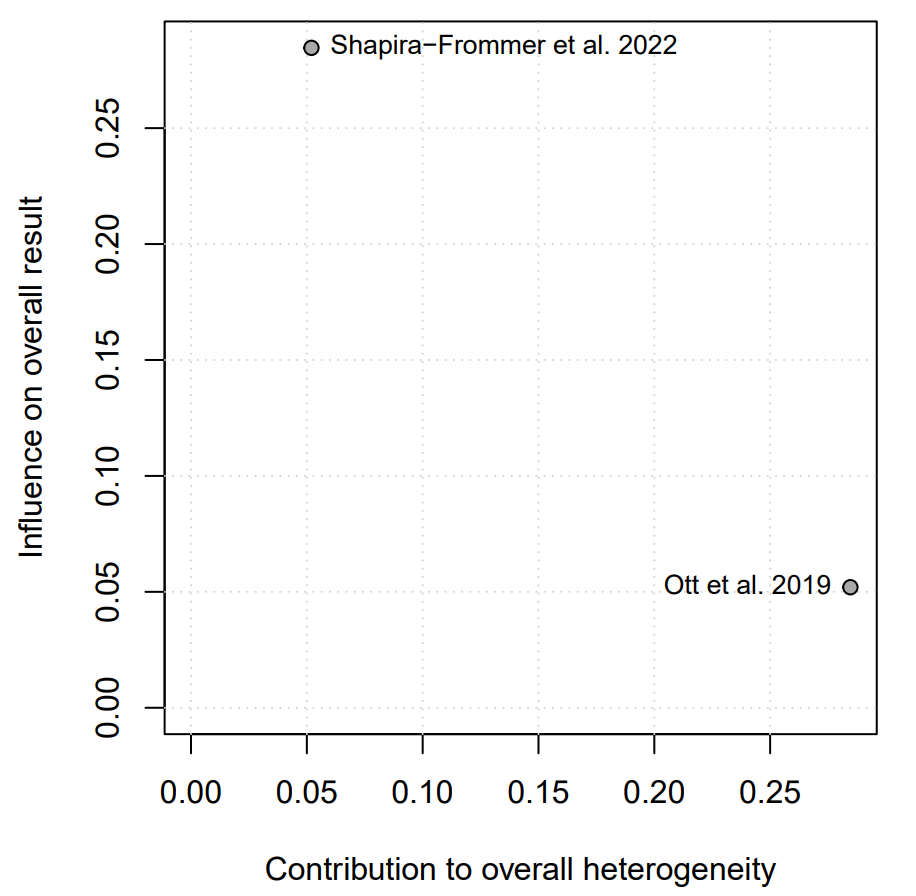


**Figure S28.** Baujat plot, PFS, ITT, 12 months.


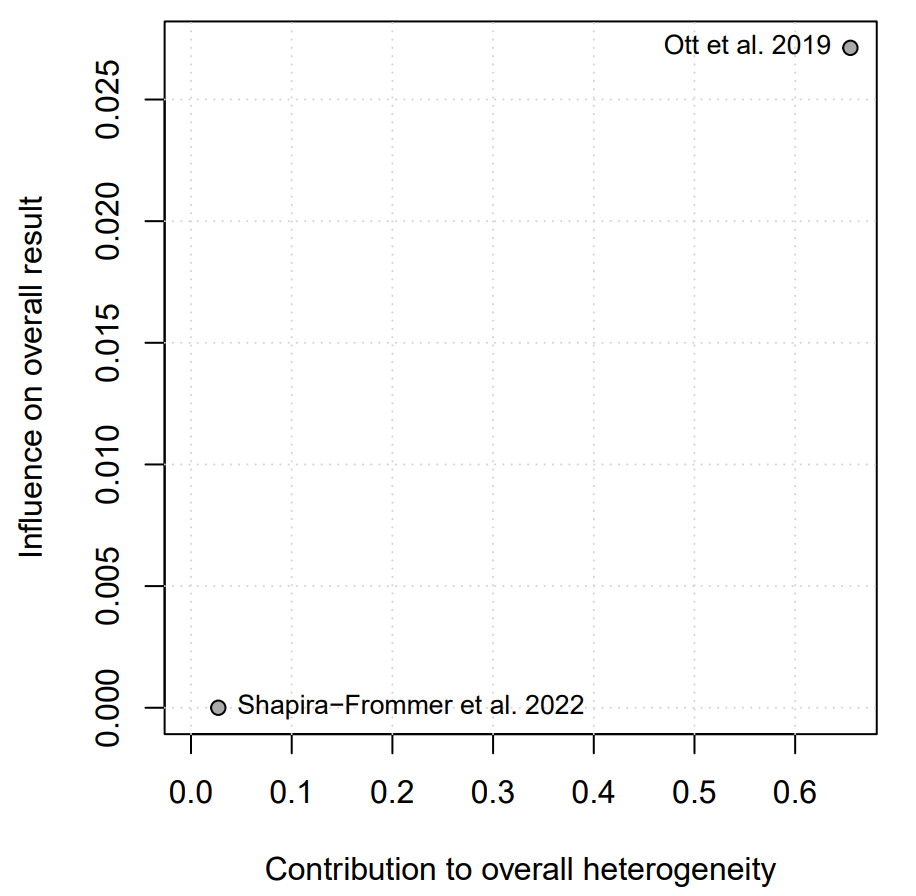


**Figure S29.** Baujat plot, PFS, ITT, 24 months.


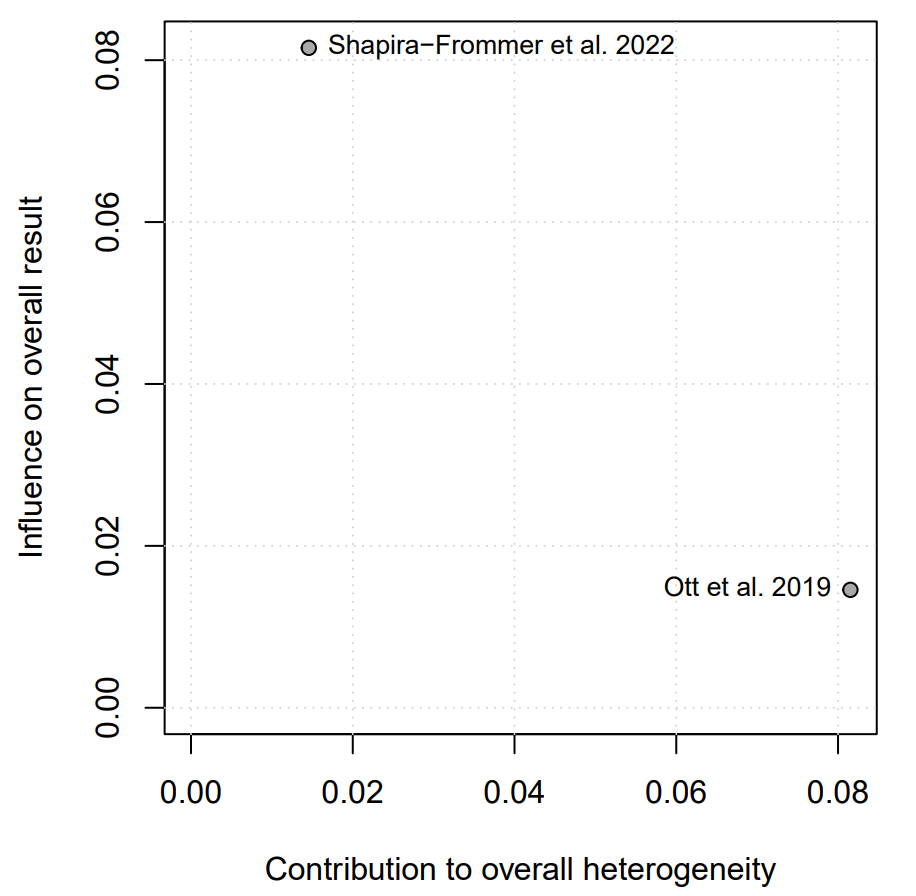


**Figure S30.** Baujat plot, PFS, PPA, 6 months.


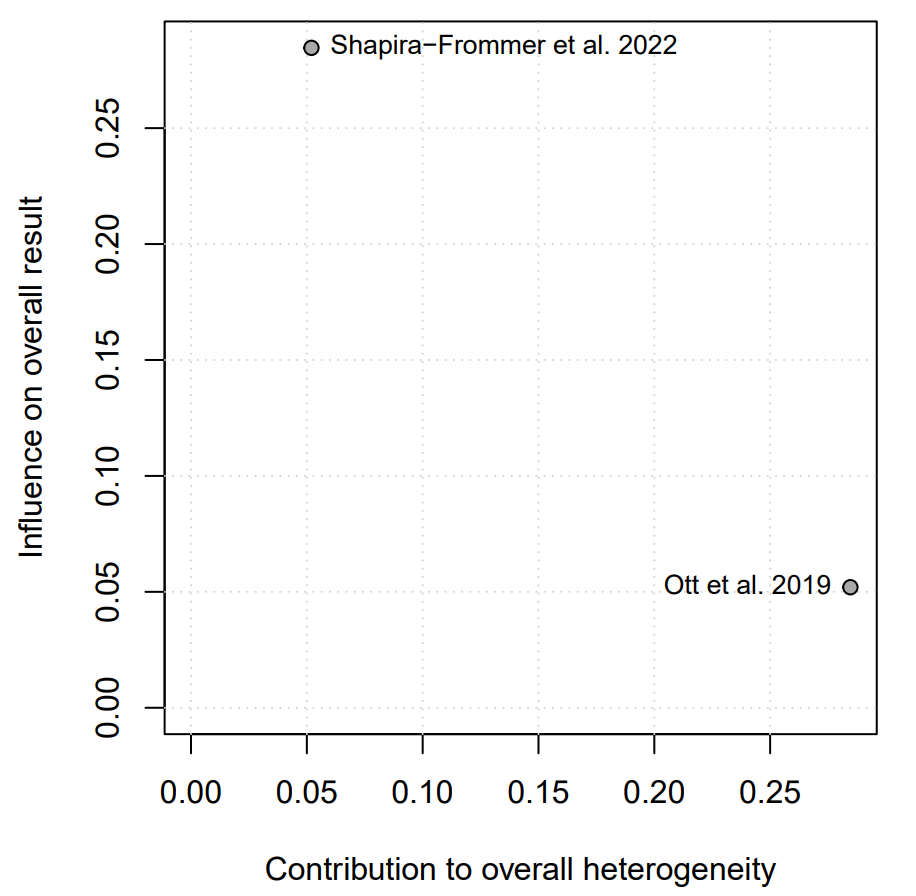


**Figure S31.** Baujat plot, PFS, PPA, 12 months.


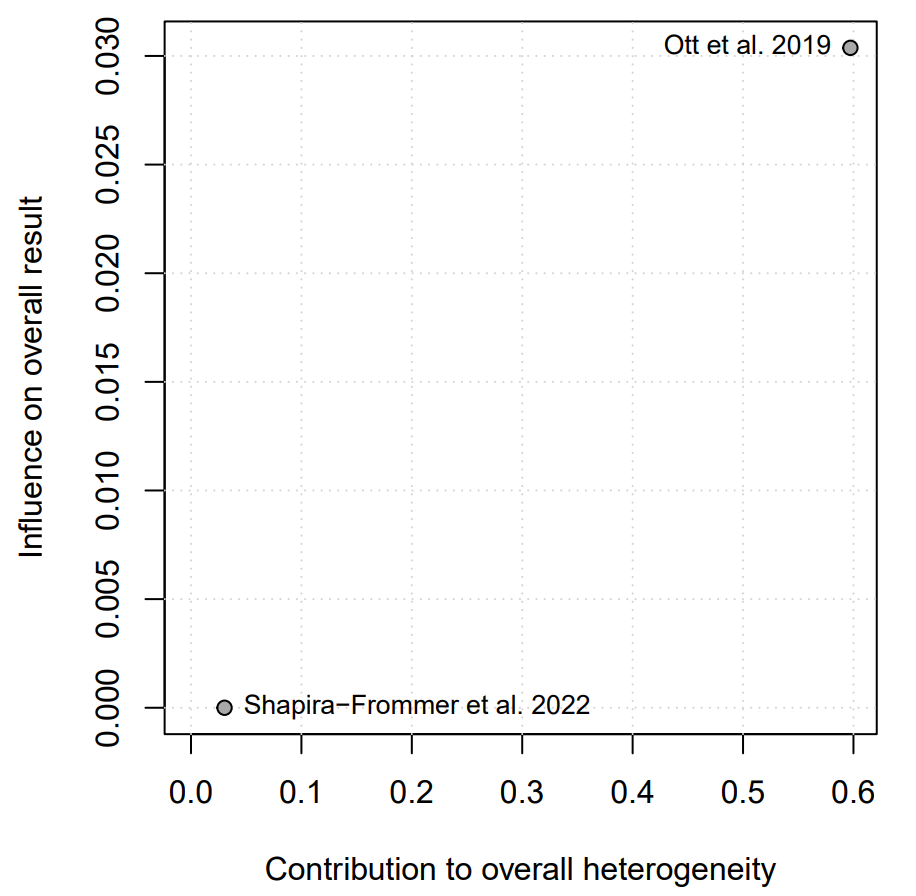


**Figure S32.** Baujat plot, PFS, PPA, 24 months.


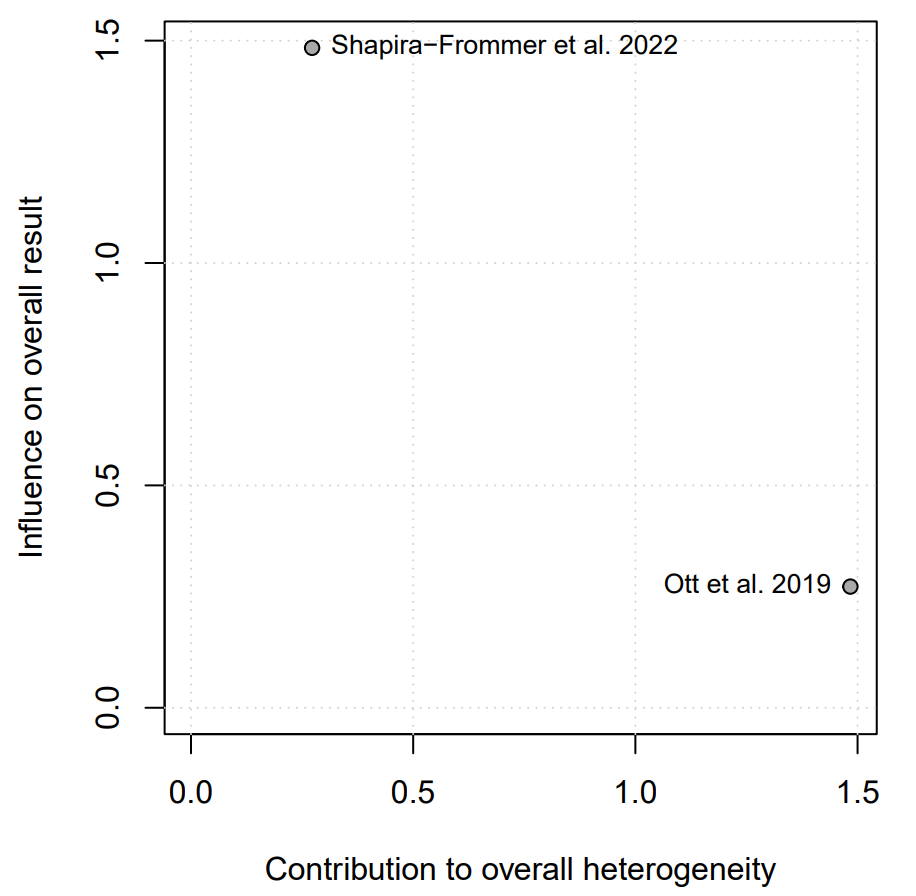


**Figure S33.** Baujat plot, OS, ITT 6 months.


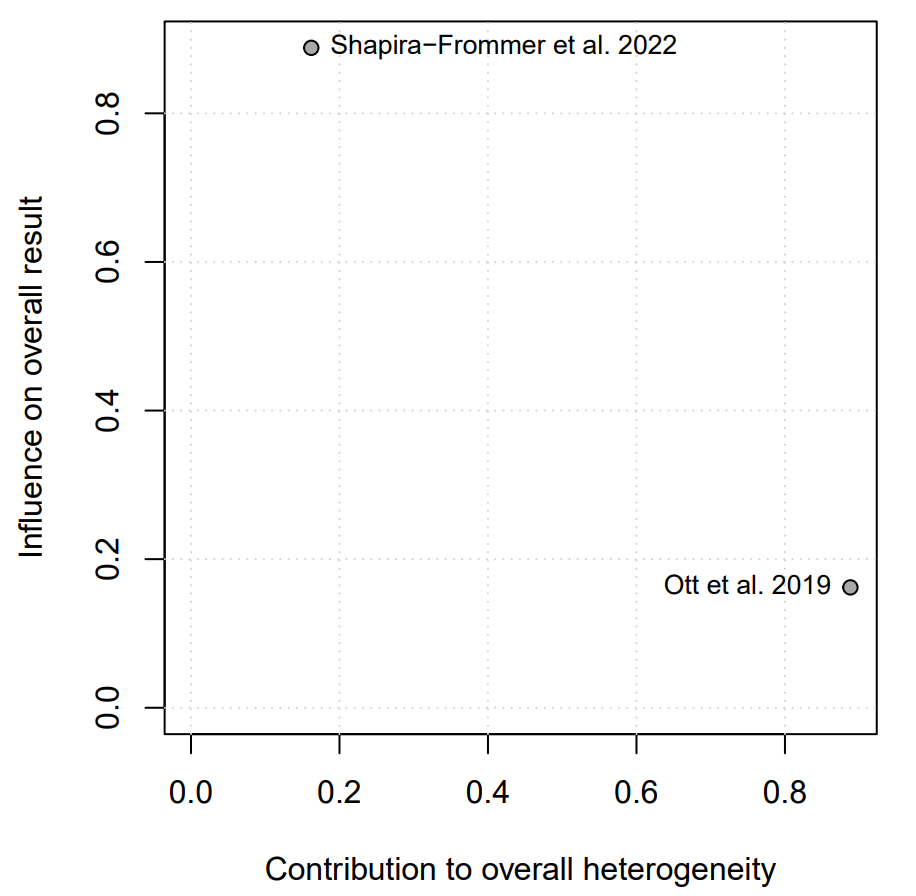


**Figure S34.** Baujat plot, OS, ITT, 12 months.


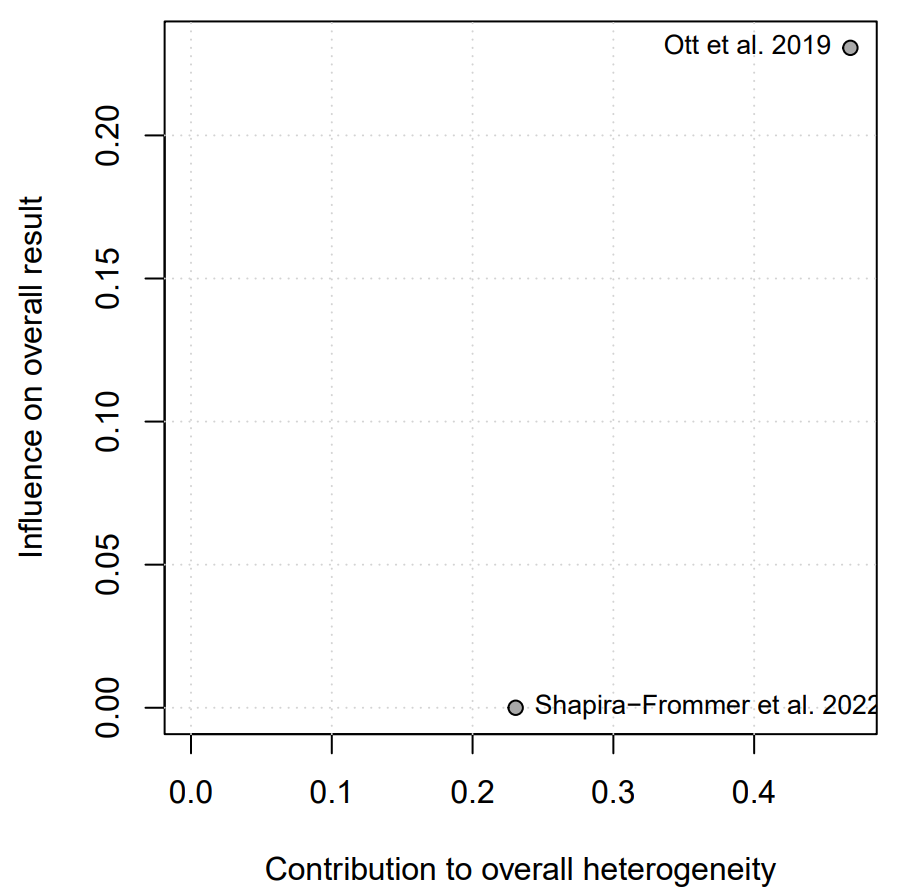


**Figure S35.** Baujat plot, OS, ITT, 24 months.


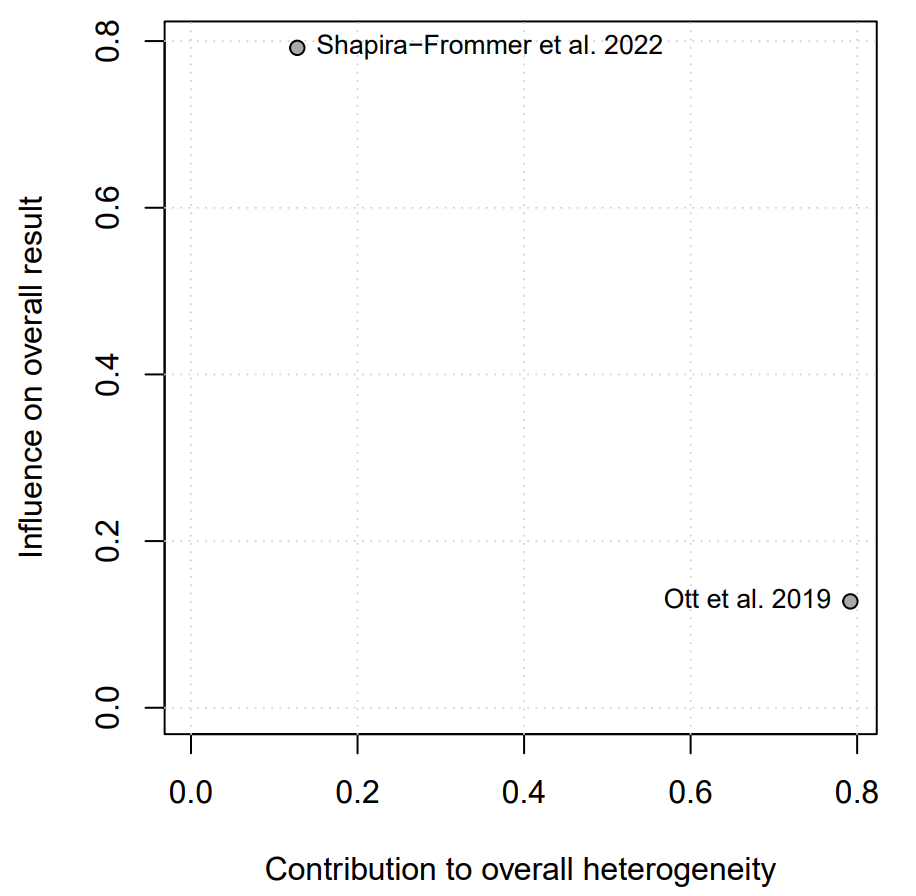


**Figure S36.** Baujat plot, OS, PPA, 6 months.


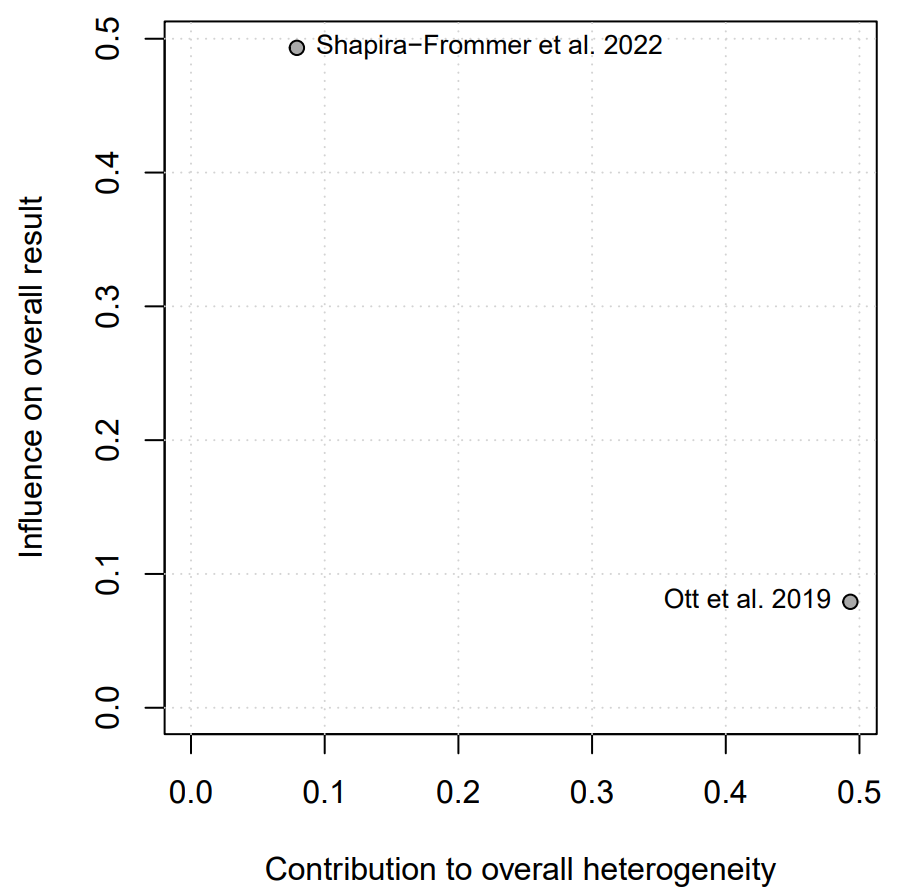


**Figure S37.** Baujat plot, OS, PPA, 12 months.


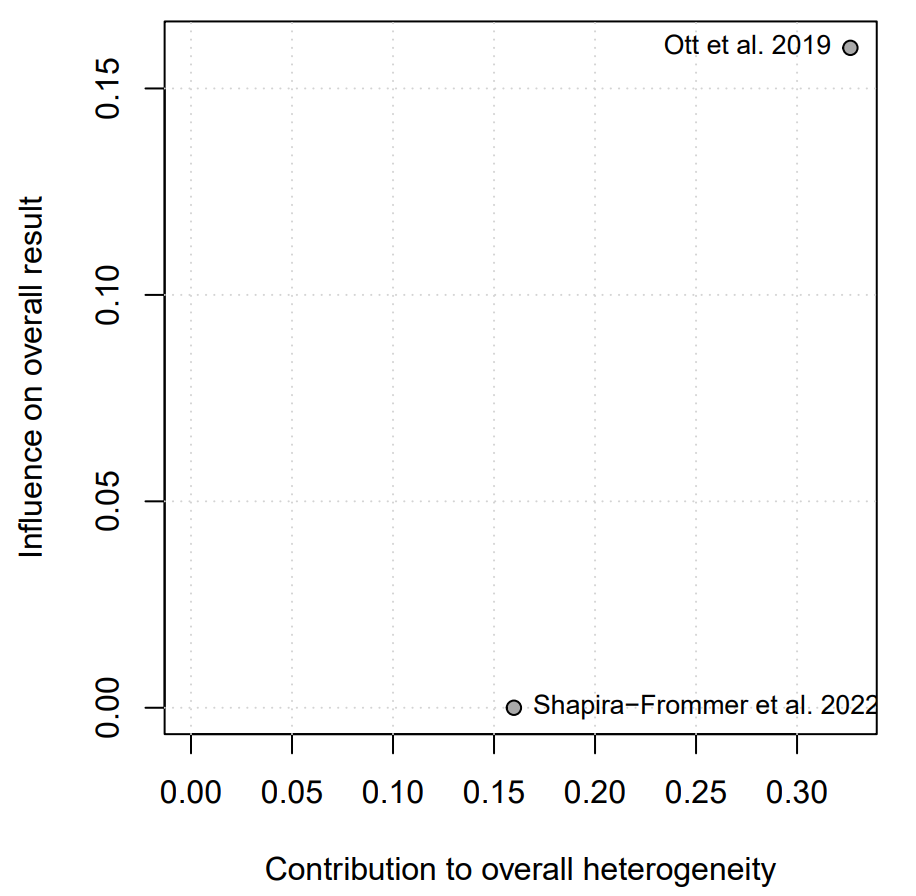


**Figure S38.** Baujat plot, OS, PPA, 24 mo.


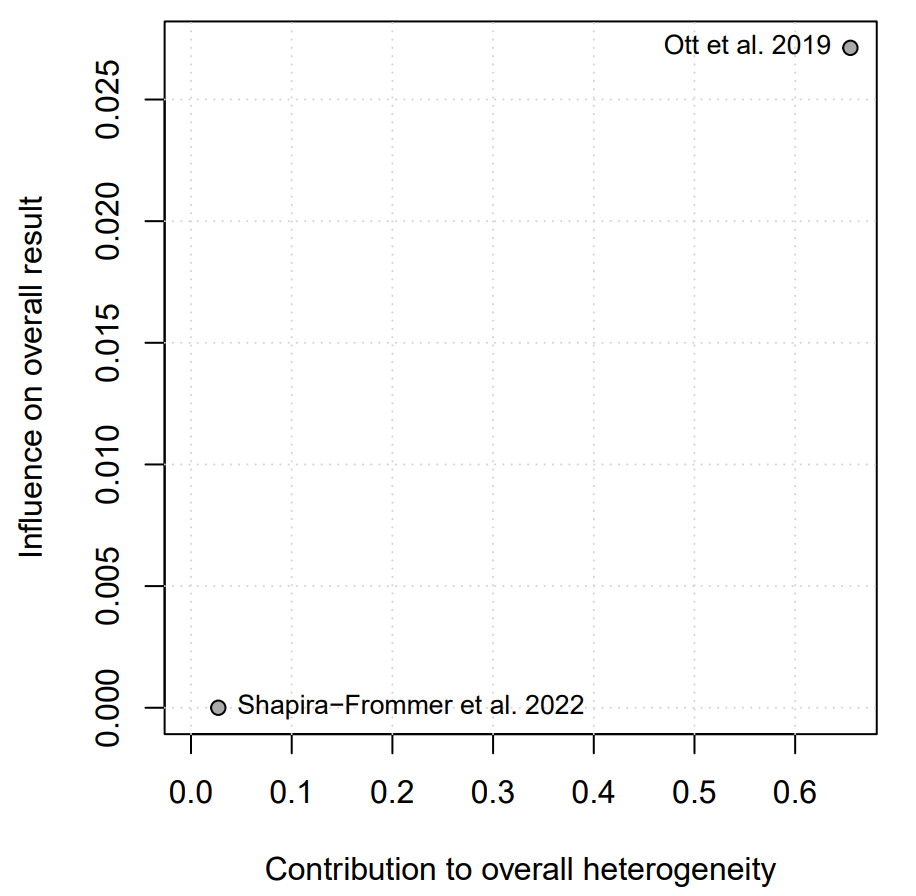


**Figure S39.** Baujat plot, Complete response, ITT.


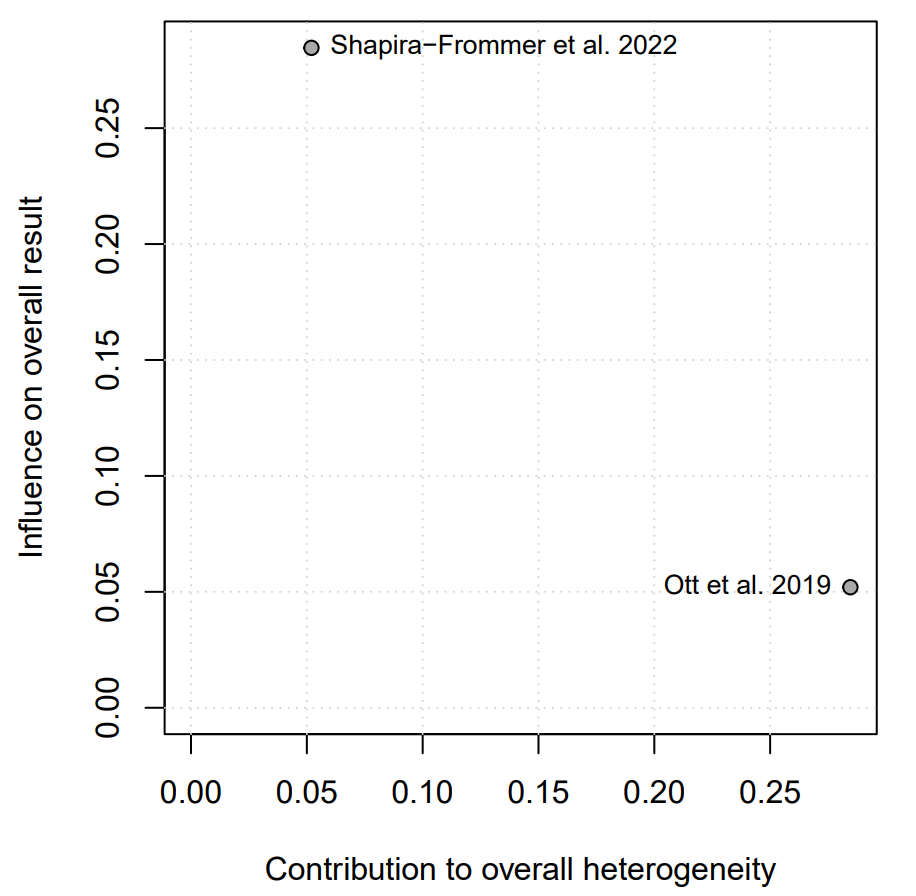


**Figure S40.** Baujat plot, Partial response, ITT.


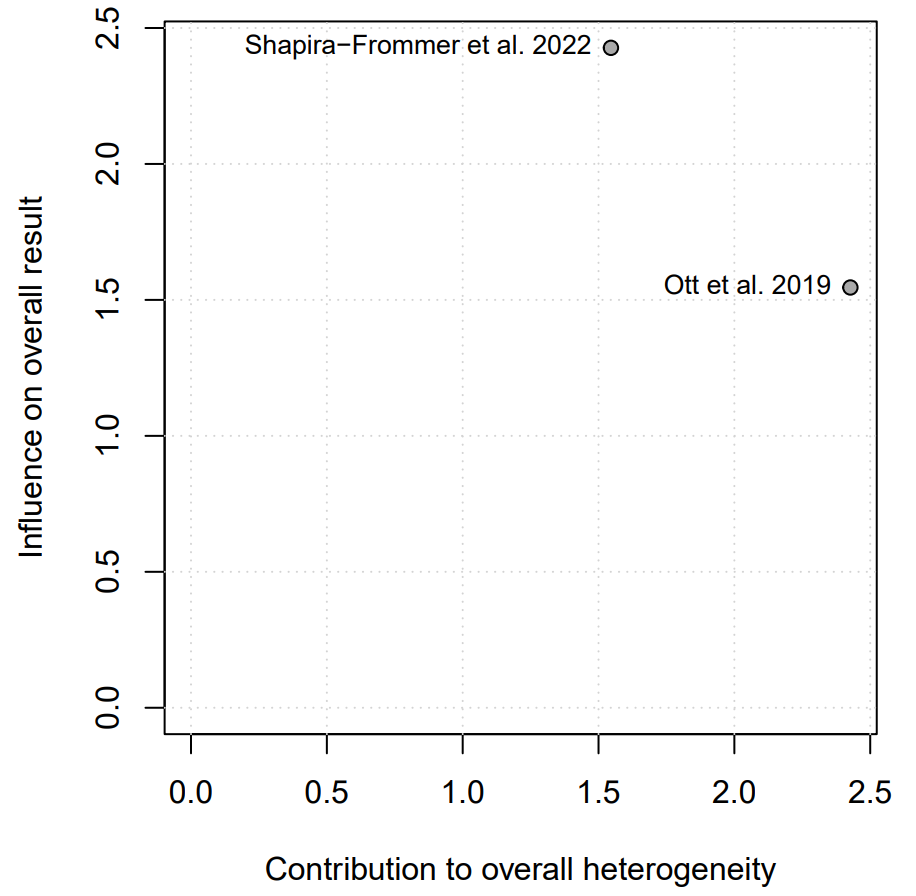


**Figure S41.** Baujat plot, Stable disease, ITT.


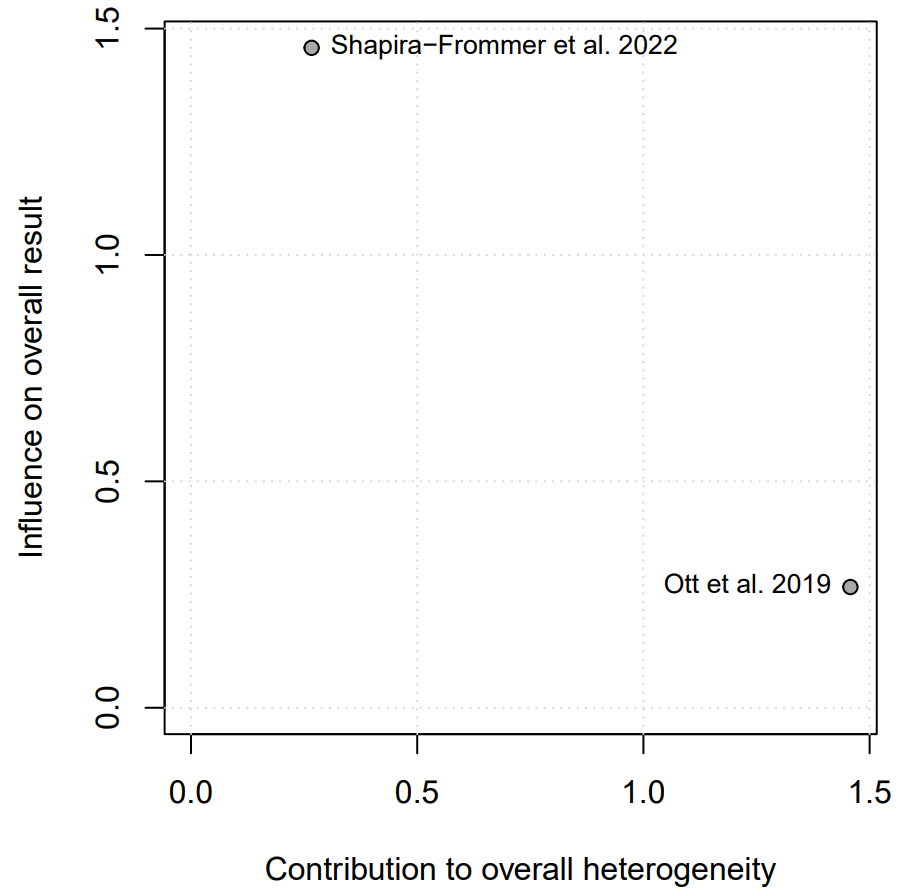


**Figure S42.** Baujat plot, Any benefit, ITT.

**
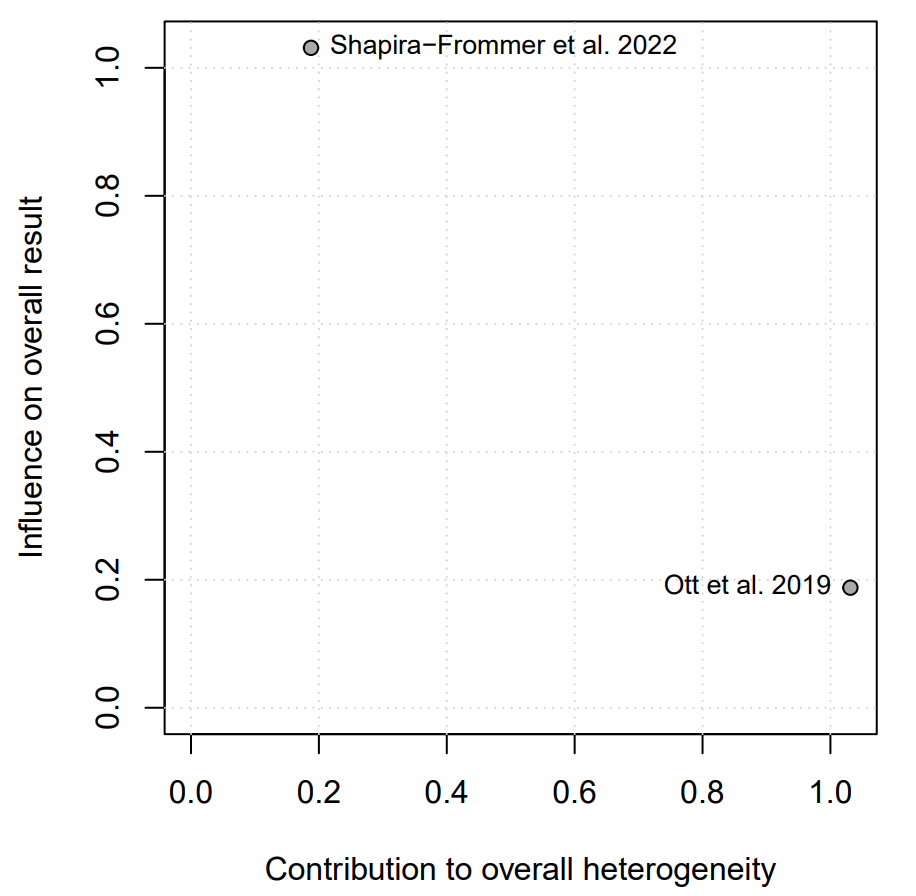
**

**Figure S43.** Baujat plot, Progressive disease, ITT.


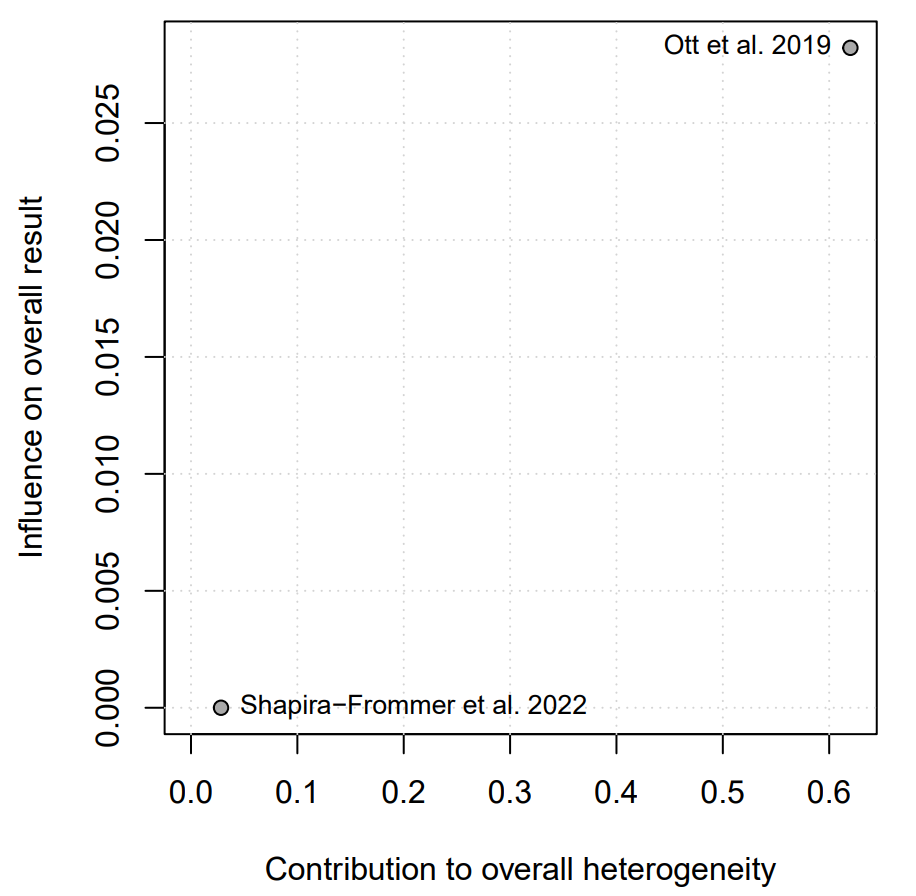


**Figure S44.** Baujat plot, Complete response, PPA.


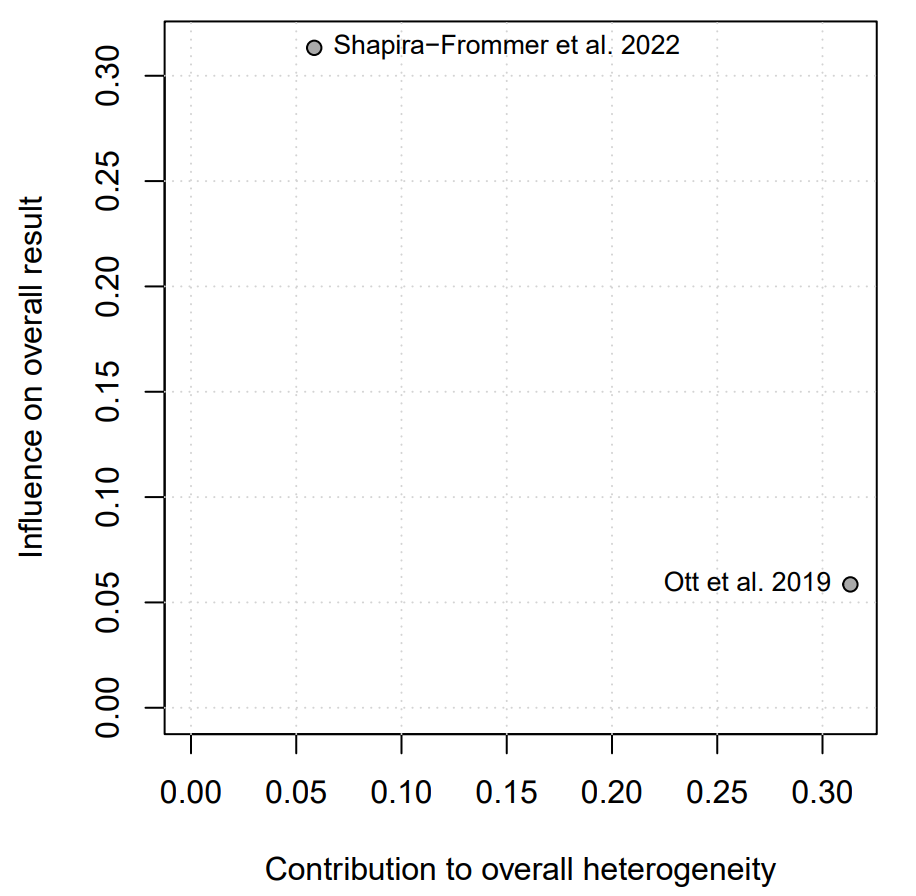


**Figure S45.** Baujat plot, Partial response, PPA.


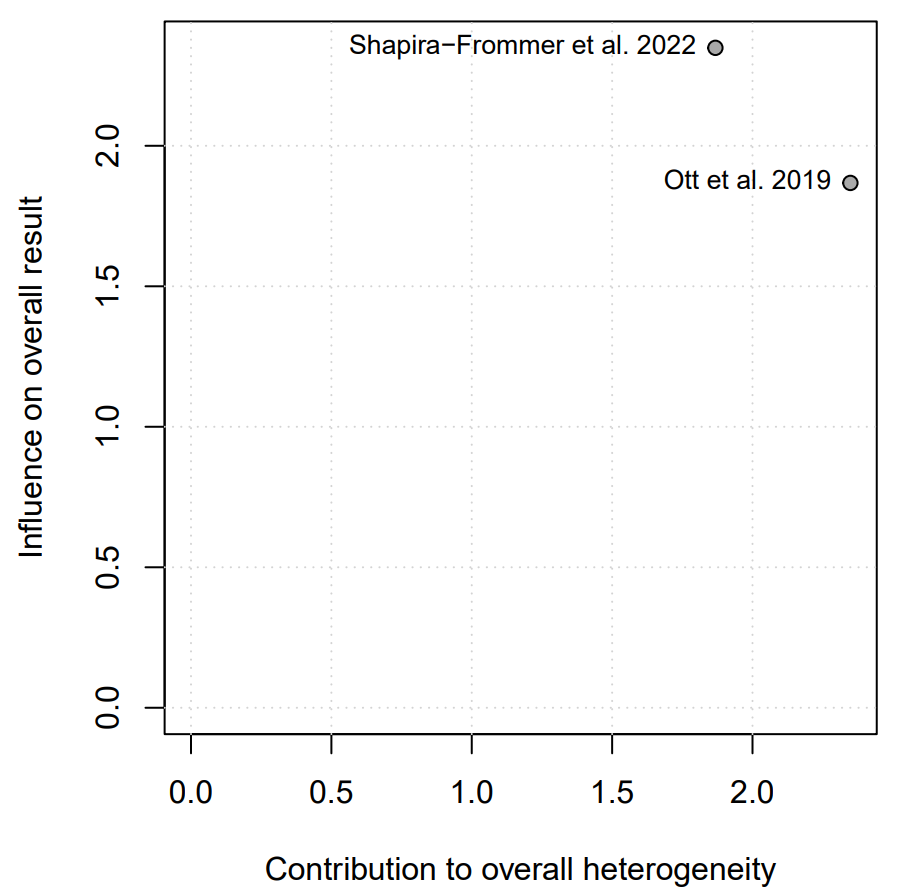


**Figure S46.** Baujat plot, Stable disease, PPA.


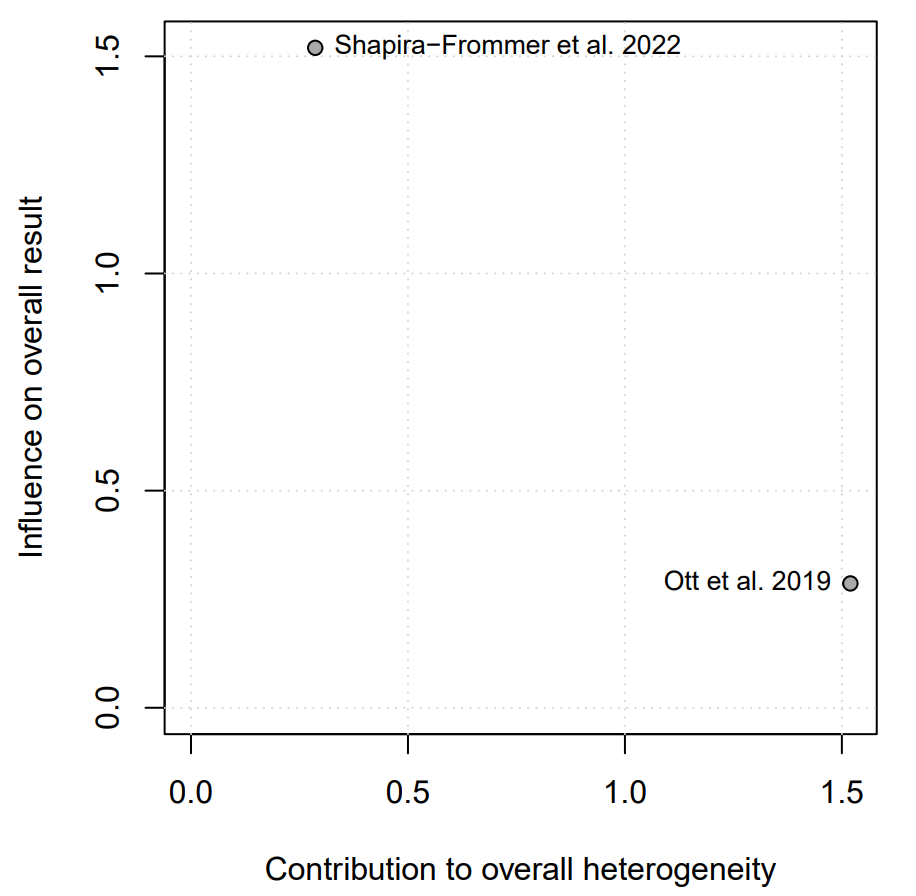


**Figure S47.** Baujat plot, Any benefit, PPA.

**
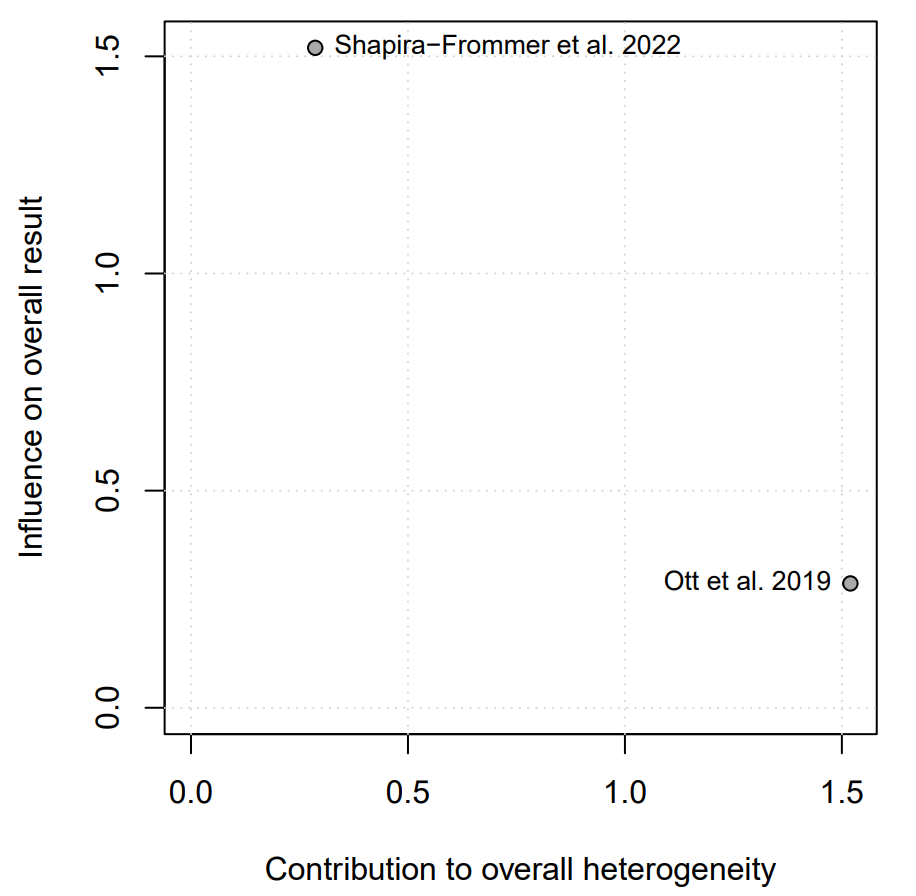
**

**Figure S48.** Baujat plot, Progressive disease, PPA.
